# Probing individual quantum emitters in bulk semiconductors via photonic nanojets

**DOI:** 10.1126/sciadv.aea5936

**Published:** 2026-05-20

**Authors:** Behrooz Semnani, Sai Sreesh Venuturumilli, Mohammad Soltani, Pratik Adhikary, Abdolreza Pasharavesh, Nikolay Videnov, Paul Anderson, Supratik Sarkar, Vinodh Raj Rajagopal Muthu, Michal Bajcsy

**Affiliations:** ^1^ Institute for Quantum Computing (IQC), University of Waterloo, Waterloo, N2L3G1, Canada.; ^2^ Department of Electrical and Computer Engineering, University of Waterloo, Waterloo, N2L3G1, Canada.

## Abstract

Solid-state quantum emitters serve as fundamental building blocks for quantum information processing, quantum telecommunication, and quantum-enhanced sensing, particularly at the nanoscale. However, the high refractive index of the emitters’ host materials often result in low-efficiency collection of the photons generated by the emitters. In addition, isolating individual quantum emitters generally requires high-purity samples and precise defect implantation, adding to the fabrication complexity. In this work, we use free-form topology optimization to design broadband monolithic photonic structures within high–refractive index materials hosting a relatively dense ensemble of randomly distributed quantum emitters. Fabricated via standard top-down patterning techniques, these inverse-designed nanostructures generate tightly confined photonic nanojets, which in turn enable selective excitation of individual emitters and improve photon extraction efficiency. The optimized geometries also substantially suppress background photoluminescence from near-surface optically active defects and from randomly distributed emitters in the bulk, boosting the signal-to-noise ratio. We demonstrate this paradigm using negatively charged nitrogen vacancy (NV^−^) centers in a low-cost diamond sample at room temperature as a case study, achieving selective single emitter excitation with a 10-fold power enhancement and more than 15-fold improvement in the photon extraction efficiency of photoluminescence collection in confocal microscopy. Beyond NV centers and other diamond-embedded quantum emitters, the use of inverse-designed structures generating subwavelength photonic nanojets is applicable to other semiconductor materials containing emitters and can be seamlessly generalized to fiber-integrated platforms.

## Teaser

Photonic nanojets act as optical spotlights, isolating color centers and efficiently extracting their photons.

## INTRODUCTION

Optically active defects in wide-bandgap semiconductors (*1*, *2*), such as diamond, can serve as stable quantum emitters and be used as single-photon sources and optically addressable spin qubits (*3*, *4*). For instance, nitrogen vacancy (NV) centers in diamond allow high-fidelity initialization, coherent control, and optical readout of electron and nuclear spin states with exceptional coherence times (*5*), even at room temperature. This makes them suited for quantum memory architectures (*6*) and ultrasensitive detection of weak magnetic fields down to the single nuclear spin level (*7*). However, efficiently addressing individual quantum emitters and extracting single-photon flux is crucial for such applications. In particular, enhanced collection efficiency improves entanglement rates for quantum communication and boosts read-out fidelity for quantum sensing (*8*, *9*).

Selective excitation of individual color centers typically requires a combination of highly localized optical excitation with a sparsely distributed defect population in high-purity samples. Major successful demonstrations of nonclassical emission from defect centers in the past relied on quantum-grade samples with highly controlled implantation of defect centers (*10*–*18*). At the same time, efficient photon extraction from isolated defect centers in a host material presents another major challenge. Many semiconductors hosting optically active defects have large refractive index at the operating optical wavelengths, such as *n* ≈ 2.4 for crystalline diamond, *n* ≈ 2.6 for silicon carbide, and *n* ≈ 3.4 for single-crystal silicon, resulting in low photon out-coupling due to total internal reflection at the air-semiconductor interface. Hence, significant research efforts have been focused on developing highly efficient optical interfaces, with diamond as a leading platform (*19*). Central to these efforts is the physical isolation of the optically active defect centers within the crystal lattice, such as—in the case of diamond—using nanocrystals (*20*, *21*) or thinning the sample down to hundreds of nanometer scales (*10*). However, these methods often face scalability limitations, posing challenges for large-scale device fabrication. For instance, nanocrystals usually rely on random techniques (drop/spin-casting) or alignment-sensitive methods (pick-and-place) to position the nanocrystals for emitter–optical field coupling. Top-down techniques, such as fabricating photonic nanowires (*13*, *22*, *23*) and nanobeams (*24*), isolate the emitters through transverse physical etching. However, this also brings the emitter close to etched surfaces, which can increase spin decoherence (*25*). While efficient devices can be sculpted from bulk diamond by thinning the substrate (*10*), these methods still necessitate delicate layer transfer, limiting their suitability for scalable manufacturing.

Alternatively, techniques such as grayscale lithography combined with resist reflow or ion beam milling have been used to produce smooth, spherical solid-immersion lenses with engineered dimensions (*14*). Going beyond conventional optical elements, Huang *et al.* (*26*) demonstrated a two-dimensional (2D) monolithic flat solid immersion platform that uses a metasurface design concept. However, these metasurfaces are usually limited in bandwidth and therefore suited for emitters with dominant zero-phonon emission. In general though, approaches based solely on ray optics inherently result in the loss of ~50% of the emitter’s radiation to the back of the substrate. Thus, other design techniques based on evanescent coupling to metallic nanoantennas (*27*) have also been explored to enhance the collection of backward-emitted radiation.

Here, we present a highly efficient design concept using adjoint optimization (*28*, *29*) to effectively excite defect centers using highly localized photonic nanojets (PNJs) (*30*, *31*) in bulk diamond as a case study. To demonstrate its effectiveness, we use high-pressure high-temperature (HPHT) synthetic diamond samples (type Ib) with uncontrolled distributions of NV centers. PNJs are highly confined optical beams with subwavelength transverse localization that propagate over several wavelength ranges by incorporating evanescent modes that interfere with propagating waves (*30*). The localization mechanism arises from edge diffraction, making PNJs inherently nonresonant and broadband and thus capable of capturing the entire fluorescence bandwidth of NV centers at room temperature. Moreover, by using near-field rather than far-field focusing, PNJs redirect backward fluorescence emission from NV centers and enhance light extraction by tailoring the angular emission of a single oriented emitter. In contrast to previous free-form designs (*29*, *32*), our approach uses near-field focusing within monolithic platforms, effectively eliminating the need for hybrid material transfer. The design concept is based solely on index contrast with air; therefore, the approach can be readily extended to various solid-state quantum emitters across a range of host materials. These include other optically active defects in diamond (*1*, *19*), silicon carbide (*33*, *34*), color centers in silicon (*18*, *35*), quantum dots embedded in III-V heterostructures (*2*), and rare earth ion-doped solids (*36*).

## RESULTS

### Design, concepts, and device fabrication

**Fig. 1.**
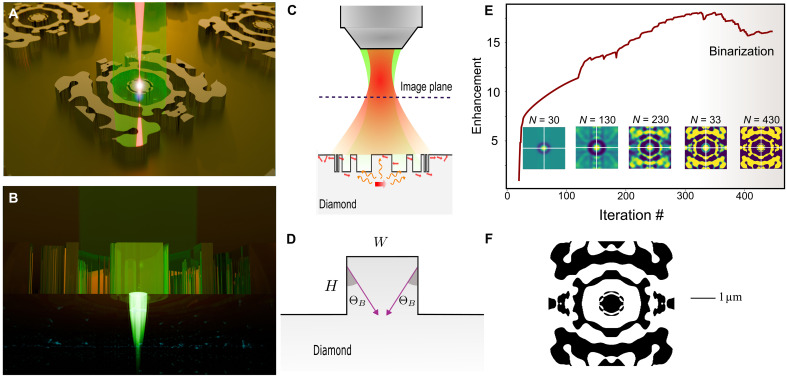
Free-form topology optimization of the photon extractor. (**A**) Illustration of the monolithically etched photon extractor in bulk diamond, optimized within a 5 μm–by–5 μm domain with a 600-nm etching depth. Extractors are arranged in a square array with an 8-μm pitch, generating PNJs under 532-nm excitation. The broadband PL by optically isolated NV centers (shown by red) is efficiently coupled out. (**B**) Illustration of the excitation field confinement. The PNJ addresses individual NVs at a 100-nm depth below the surface. (**C**) To reduce background fluorescence, the PL from the nanojet-excited NV center(s) is tightly focused into a Gaussian profile with a 0.5-μm beam waist at 2 μm above the surface. The height offset reduces collecting PL from shallow-depth emitters. (**D**) Step-like topology used as the initial structure for optimization to excite a PNJ via edge diffraction. The angle Θ*_B_* represents the approximate orientation where nanojet lobes (the edge jets) form due to diffraction. (**E**) Inverse design convergence profile, starting with a subwavelength pillar. The curve shows the enhancement of power coupling to the selected Gaussian profile. The optimization involves grayscale and binarization of the refractive index profile. (**F**) Final optimized pattern.

The topology optimization of monolithic photon extractors primarily targets the maximization of photon out-coupling efficiency by exploiting near-field interactions. Furthermore, the design is tailored to enhance contrast against background photoluminescent emission, thereby ensuring nonclassical photon statistics. In particular, the structure is optimized for a confocal setup, where the broadband photoluminescence (PL) is collected using a high numerical aperture (NA) objective lens. We designed the structure to meet three key criteria. First, a dipole source is positioned 100 nm below the structure representing an isolated emitter to ensure near-field coupling and efficient excitation of the confined mode. Second, to maximize the collection efficiency of NV fluorescence by a high NA objective while minimizing the computational domain, the PL emission is optimized to overlap with a tightly focused Gaussian mode close to the diffraction limit (waist diameter of ∼0.5μm) at a height of 2 μm above the surface (Fig. 1C). This height offset is determined according to the axial (depth) resolution of the high NA confocal microscope, that is, $\Delta z≲2\lambda /{\text{NA}}^{2}$, where λ is the upper wavelength of the PL and NA is the numerical aperture of the objective lens (*37*), effectively suppressing unwanted PL contributions from surface emitters and enhancing the purity and efficiency of the collected signal. Third, the structure is optimized for excitation and collection in the x-polarization state, improving signal-to-background contrast. Despite NV centers in (100)-oriented diamond exhibiting dipole moments lying on the (111) plane, polarization selectivity enhances the signal-to-noise ratio by channeling the emission into the x-polarization state, thereby suppressing the background PL with random polarization.

Pairs of forward/adjoint simulations were conducted iteratively to optimize photon extraction (*28*). The optimization region was selected as a 5 μm–by–5 μm area with an etching depth of 600 nm. The forward simulation used a broadband, x-polarized electric dipole positioned 100 nm beneath the etched diamond surface. This dipole emitted light spanning the fluorescence band of NV centers along with the above-band spectrum, ensuring precise field localization during excitation. The adjoint simulation was driven by a tightly focused Gaussian mode located at 2 μm above the diamond surface (see the Supplementary Materials, fig. S2). The confinement of the Gaussian mode is dictated by the resolution of the confocal microscope, maximizing PL collection at a single spot during confocal scanning.

By Lorentz reciprocity in linear reciprocal media, maximizing the coupling of dipole radiation into this Gaussian mode is formally equivalent to maximizing the electric field at the location of the dipole when the structure is illuminated by the same Gaussian profile. Therefore, the topology optimization simultaneously promotes NA-limited photon extraction efficiency and excitation field localization. At the same time, excitation of a PNJ is primarily driven by the initial geometry established at the onset of the optimization process. Since topology optimization is inherently a gradient-based technique, the choice of initial geometry influences the basin of attraction and thereby the final structure to which the algorithm converges. To generate PNJs in the initial geometry, we leverage the principle of edge diffraction in stepped-light topologies, beginning with a pillar-like seed structure (*38*). The dimensions of the pillar are selected on the basis of edge diffraction approximation (*38*), where nanojet formation arises from constructive interference between diffracted and refracted light within the dielectric material. As shown in Fig. 1D, the diffracted light constructively interferes with the refracted light at an angle $\Theta_{B}\approx \frac{1}{2}\left(\frac{\pi }{2}-\theta_{\text{TIR}}\right)$ relative to the air-dielectric interface on the stepped geometry (*38*), forming so-called edge jets. Here, θ_TIR_ denotes the angle of total internal reflection. The edge jets are directed toward a common focal point. Accordingly, the initial diameter of the pillar is determined using *W* ≈ 2*H* tan (Θ*_B_*), ensuring the formation of a nanojet just below the pillar. For the case of the diamond pillar, where Θ*_B_* ≈ 32°, an aspect ratio of *W*/*H* ≈ 1.25 effectively drives the nanojet formation. The simulation results illustrating nanojet formation in the initial step-like geometry are presented in the Supplementary Materials. Figure 1E shows the convergence profile over 420 iterations and the optimized pattern, respectively. The algorithm uses grayscale and binarization phases to achieve the optimized index profile shown in Fig. 1F.

**Fig. 2.**
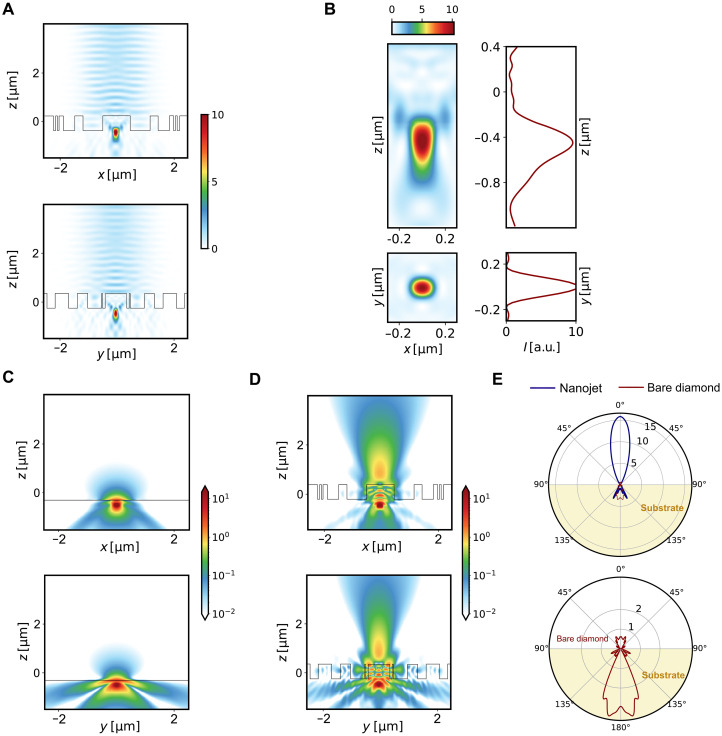
Simulation results. (**A**) Field profile in the excitation mode with the structure illuminated by a 532-nm Gaussian coherent laser beam with a 4-μm beam waist. The field profiles are shown in the *xz* and *yz* planes. (**B**) Formation of nanojets just below the structure in the bulk. The nanojets provide subwavelength focusing while extending the beam over several wavelength scales. (**C**) PL radiation profile for a bare diamond with no structure on the surface, showing radiation from near-surface emitters. (**D**) PL radiation profile from the nanojet-excited NV centers. Only the field profile at the NV^−^ peak emission wavelength, i.e., 680 nm, is shown here, but the structure is fairly broadband (see the Supplementary Materials). The extractor significantly enhances upward radiation, coupling it into a Gaussian profile at 2 μm above the sample. (**E**) Directivity pattern of the PNJ. Comparison between the radiation pattern of NV centers located near the surface of bare diamond and that of the inverse-designed nanojet structure. The inverse-designed structure couples light preferentially upward, yielding at least a 10-dB enhancement in directivity and substantially suppressing backward radiation into the substrate. a.u., arbitrary units.

The optimized structure was simulated using Lumerical finite-difference time-domain (FDTD) under two regimes: the excitation regime at 532-nm illumination and the PL collection regime at 680 nm, corresponding to the NV fluorescence peak at room temperature. Figure 2 (A and B) illustrates the formation of a PNJ during 532-nm laser excitation. This nanojet is tightly focused by leveraging near-field interactions coupled with propagating far fields and propagates along the *z* axis, maintaining its focus across multiple wavelength scales (refer to Fig. 2B). The efficacy of the structure in photon outcoupling was evaluated through dipole radiation simulations at the peak wavelength of 680 nm performed both with and without the inverse design structure. Figure 2 (C and D) reveals that, for bare diamond, the high refractive index of diamond restricts light emission to narrow angles, allowing only a small fraction to escape the bulk while ~94% of the radiation is reflected back into the substrate due to total internal reflection. In contrast, the topology-optimized structure significantly enhances photon outcoupling and suppresses backward radiation into the substrate. This improvement is depicted in Fig. 2E, showcasing the radiation pattern of the NV center positioned at the nanojet. The front-to-back ratio reaches up to 12 dB, highlighting the significance of near-field interactions for enhancing optical performance. To highlight the broadband response of the structure, emission patterns of the structure at multiple wavelengths across the fluorescence band of NV centers are presented in the Supplementary Materials.

### Experimental results

**Fig. 3.**
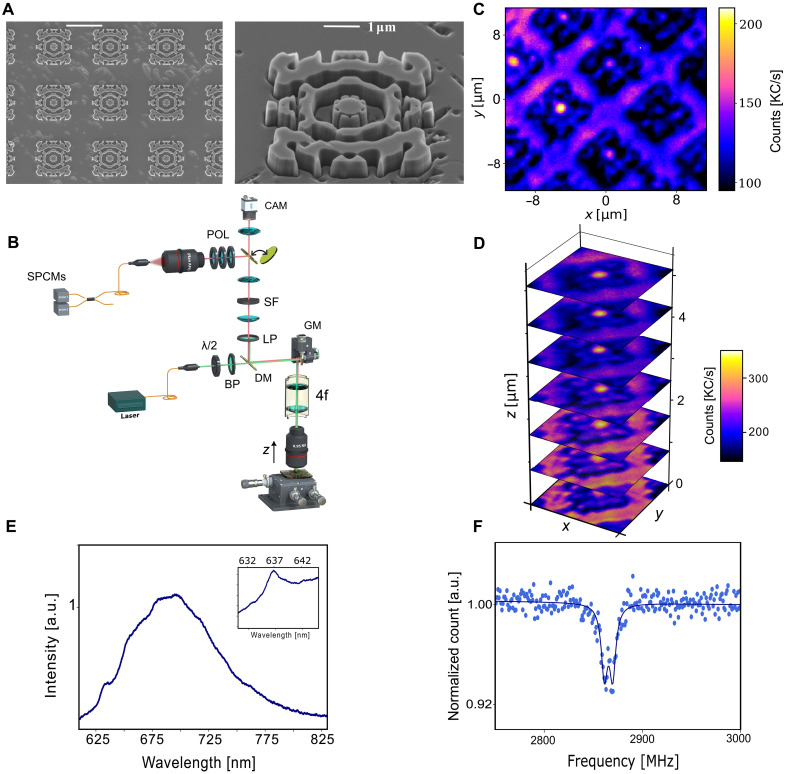
Photoluminescent confocal microscopy of the PNJ extractors. (**A**) SEM images of the fabricated device in HPHT diamond samples. The structures are fabricated as rectangular arrays with an intradevice spacing of 8 μm. The sample is etched to a depth of 600 nm. (**B**) Experimental setup. An air objective lens with NA = 0.95 is used to focus the pump green light onto the sample and collect emitted PL photons. λ/2, half-wave plate; DM, dichroic mirror; CAM, CMOS camera for basic imaging. The other component labels and the components’ functions are explained in the 'Measurement procedure’ section. (**C**) Confocal microscope image of a square array of inverse design structures at a height offset of 2 μm relative to the image plane. Dark and dark blue regions correspond to nanojets without correlated embedded NV centers and inverse structures containing weakly coupled NV centers, respectively. Structures with an NV^−^ center strongly coupled to a nanojet and acting as a single-photon source with the best performance appear as yellow spots in this image due to their high photon count rates. (**D**) Height offset scans of PL scans of the brightest structure. (**E**) PL spectrum of photons collected from a nanojet excited NV center, showing the NV center zero-phonon line at 637 nm and the phonon sideband from 640 to 780 nm. (**F**) ODMR signal associated with the *m_s_* = 0 to *m_s_* = ±1 transition without external magnetic field. The presence of ODMR splitting at 2.87 GHz can be attributed to strain and internal electric field due to surrounding nitrogen irons in the crystal of HPHT diamond.a.u., arbitrary units.

The topology-optimized structure shown in Fig. 1 was fabricated using electron beam lithography (EBL), followed by a lift-off process and reactive ion etching (RIE) with oxygen plasma. To achieve smooth vertically etched sidewalls, both the hard mask and the etching parameters were optimized in several iterations. The scanning electron microscopy (SEM) images of the fabricated device are shown in Fig. 3A. A detailed description of the fabrication steps is provided in Materials and Methods. The diamond jet structures were optically characterized using a custom-built laser scanning confocal microscope equipped with a 0.95 NA dry objective lens, a 4f relay optics, and scanning galvo mirrors (refer to Fig. 3B). A continuous fiber-coupled 532-nm laser was used to excite the vacancy centers. To remove the pump laser from the collection path and also to suppress fluorescence contributions from the neutral NV centers and Raman signals from the diamond crystal, a long-pass 650-nm filter was used. In addition, to achieve better contrast against the background, the input laser was polarized along the principal axis of the device, consistent with the design specifications. High-throughput coarse scannings were initially conducted to rapidly identify devices with the highest photon count rates from large arrays. The confocal setup incorporates a self-correcting feedback mechanism to compensate for drift over time, enabling fluorescence measurements over extended periods. More detailed description of the characterization procedure is discussed in Materials and Methods. As highlighted earlier, the confocal scan leverages an offset plane to maximize PL collection from NV centers that sit within the nanojet mode, effectively reducing background PL. The PL map in Fig. 3C reveals a pronounced PL contrast in selected jet structures. Furthermore, we performed PL scans at multiple height offsets, as shown in Fig. 3D where *z* = 0 corresponds to the diamond surface plane from which light is collected. A height offset of ~3.2 μm yields the highest contrast, slightly deviating from the simulation results, which predict a maximum at a slightly lower offset of ~2 μm.

We confirmed dominant PL emission from negatively charged NV centers by measuring the fluorescence spectrum using a high-throughput, high-resolution Czerny-Turner spectrometer with extended integration time. The emission spectrum of the selected NV center is shown in Fig. 3E. To further investigate the characteristics of PL emission from the PNJ, the optically detected magnetic resonance (ODMR) signal was measured under microwave excitation. In the absence of an external magnetic field, electron spin coupling to nonzero spin states resulted in a single dip in the fluorescence spectrum at 2.87 GHz. However, clusters of N^+^ ions surrounding NV^−^ centers often generate internal electric fields, which can lead to splitting of the ODMR signal (*39*, *40*). The zero-field splitting is particularly pronounced in anisotropic HPHT type Ib diamond samples (*41*), which exhibit high nitrogen concentrations (~100 parts per million), with only 0.1% converted into NV centers. The ODMR splitting observed in Fig. 3F is likely a cooperative effect of both strain and internal electric fields arising from the ion concentration (*41*).

**Fig. 4.**
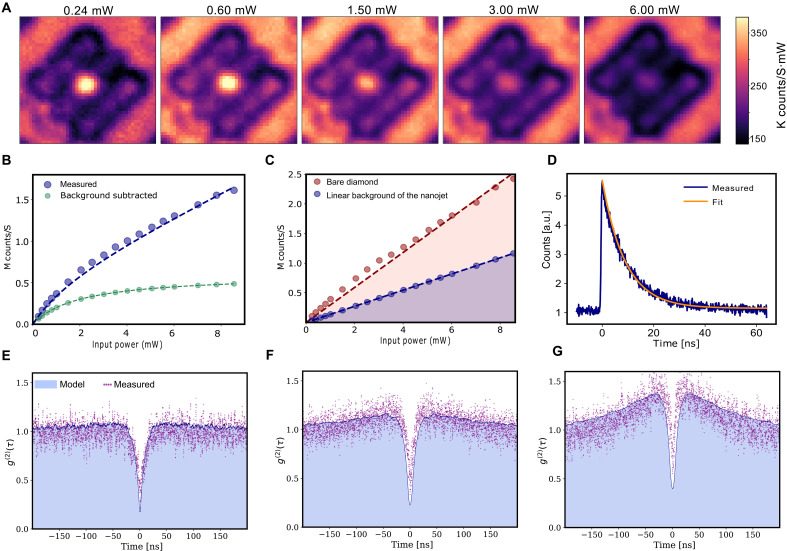
Saturation behavior and photon antibunching. (**A**) Power-normalized PL scans (i.e., PL/P_exc_) for various power levels around an extractor with embedded NV centers on the nanojet. The PNJ saturates at much lower power levels compared to the background. Laser pump powers incident on the objective are indicated. (**B**) Saturation curve for the nanojet enhanced photon extractor with embedded NV. The blue dotted curve fits the data. Background counts are evaluated by a linear fit at high power levels, and the background-subtracted curve is shown by green dots. (**C**) Red dotted line: Linear background on bare diamond *B*^Bare^, increasing proportionally with input power. Blue dotted line: Linear background noise out coupled through the nanojet *B*^Nanojet^. The ratio of these two signals defines the background suppression ratio. (**D**) Lifetime measurement with a <130-ps pulse excitation with the center wavelength of 520 nm. The fit indicates a lifetime of τ_0_ ≈ (9.5 ± 0.5) ns. (**E** to **G**) Second-order autocorrelation function *g*^(2)^ (τ) measured experimentally (purple dots) using Hanbury Brown and Twiss (HBT) setup for excitation powers of *P* = 0.3, 0.6, and 1.8 mW, respectively. The theory prediction is shown by the blue shaded regions. The marked decrease at zero time delay [*g*^(2)^ (τ) < 1/2] indicates that photons from the device are antibunched. Qualitatively different dynamics are observed at various excitation powers.

To evaluate the single-photon count rate and overall collection efficiency, saturation measurements of PL counts were conducted around the selected structure using multiple 2D PL scans at varying excitation power levels. Figure 4A presents the PL map, where counts are normalized to the excitation power. Only the bright nanojet spot reaches saturation, while the background PL intensity increases linearly with power. This observation provides additional evidence of nanojet excitation, as the strong localization of optical power causes the NV center within the jet mode to reach saturation at relatively lower excitation powers. To further quantify this, the saturation curves for the bright fluorescence spot and background contributions are plotted in Fig. 4 (B and C), respectively. The PL signal from the nanojet consists of contributions from single-photon events (S) and background fluorescence (B), which appear as a nonzero slope in the fluorescence tail of the saturation curve at higher power levels. In these cases, it is common to extract a linear background term with respect to the excitation power *P* by fitting the detected PL intensity to $C\left(P\right)=\frac{C_{\text{sat}}}{1+P_{\text{sat}}/P}+\mathrm{\alpha P}$ where the first term represents the saturable response of the emitters, while the second accounts for the overall background contribution. The background-subtracted saturation curve, shown in Fig. 4B, indicates a saturation intensity of *C*_sat_ ≈ 0.46 × 10^6^ counts per second (cps) for the selected nanojet device. The corresponding saturation power $P_{\text{sat}}^{\text{Nanojet}}$ provides a direct measure of the excitation enhancement. Since the saturation power scales inversely with the local excitation intensity, a reduction in $P_{\text{sat}}^{\text{Nanojet}}$ relative to that of bare diamond, $P_{\text{sat}}^{\text{Bare}}$, reflects the nanojet-induced enhancement of the excitation field.

The background noise at the nanojet location is suppressed by the jet-focusing mechanism, which reduces contributions from near-surface emitters. To quantify this suppression, we compare the linear background term extracted from the nanojet region, *B*^Nanojet^ = α*P*, with that measured on a bare area of the diamond, where only background fluorescence from near-surface NVs is collected, denoted by *B*^Bare^/*B*^Nanojet^. The ratio of these two linear contributions defines the background noise suppression factor, *B*^Bare^/*B*^Nanojet^ which characterizes the ability of the nanojet to reject background fluorescence. For the selected device, this ratio is ~2.5, indicating that the nanojet suppresses the linear background noise by ~60%. This value could be further improved by optimizing the device for operation at larger offset heights, where background fluorescence is less efficiently collected by the objective lens.

In addition, we experimentally measured the fluorescence lifetime of nanojet-excited NV centers using short-pulse excitation; a ~130-ps pulse laser with the central wavelength of 520 nm was used to pump the selected structure. PL decay was statistically analyzed by collecting counts over an extended period using a single-photon counting module (SPCM) and a time tagger. The decay curve was then fitted to an exponential function, revealing a decay time of $\tau_{0}∼9.5\;\text{ns}\pm 0.5\;\text{ns}$. The fluorescence lifetime of a color center determines the upper limit on the number of single photons that can be collected. Using this information, we estimate the collecting efficiency of the entire optical confocal setup, resulting in η ≈ 0.024. This value includes both the setup efficiency and the device’s collection efficiency.

To assess the possibility of Purcell enhancement, we compared the fluorescence lifetimes of near-surface emitters coupled to the nanojet photon extractor to those of shallow emitters in bare diamond. The lifetimes are indistinguishable within experimental uncertainty, corresponding to a Purcell factor of unity, in agreement with our FDTD simulations (the Supplementary Materials and fig. S4), which predict only a marginal change in the total radiated power in the presence of the nanojet. Although pronounced near-field hotspots arise from interference under external excitation, these intensity enhancements do not generally modify the local density of states across the full emission bandwidth, and therefore, the spectral-averaged spontaneous emission rate remains unchanged.

Last, we measured second-order autocorrelation statistics, i.e., *g*^(2)^ (τ), using a Hanbury Brown and Twiss (HBT) setup consisting of a fiber beam splitter followed by two SPCMs. Figure 4 (E to G) shows the background-subtracted autocorrelation statistics for three excitation powers: 0.3, 0.6, and 1.8 mW, respectively, alongside theoretical predictions obtained through a rigorous quantum model. The marked decrease at zero time delay *g*^(2)^(0) ≈ 0.17 at low excitation powers demonstrates that photons from the device are antibunched and thus dominantly coming from a single NV. Background count rates used to remove the coherent noise from the raw counts in two channels were derived from the lowest photon counts observed across the device region. Experimentally measured statistics, depicted as purple dots in Fig. 4, show agreement with theoretical estimates represented by blue shaded regions. The details of the background subtraction technique and the predictive model for antibunching statistics are provided in the Supplementary Materials. The model incorporates an equivalent three-level structure—comprising a ground state, an excited state, and a metastable shelving state. Key parameters, such as collection efficiency (i.e., η_0_) and decay rate (i.e., 1/τ_0_), were extracted from experimental data and implemented in the model (see section S3). The bunching effect observed around the clear antibunching dip at zero delay is attributed to the presence of the metastable state and associated decay pathways. At higher power levels approaching saturation of jet-excited NV centers, the coupling to the metastable shelving state becomes more pronounced, resulting in the formation of bunching shoulders at intermediate time delays in Fig. 4 (F and G).

**Fig. 5.**
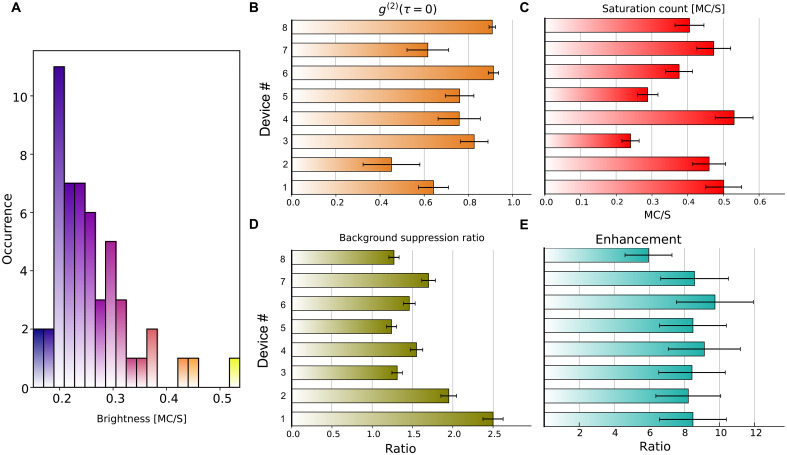
Nanojet device yield and key performance metrics. (**A**) Fluorescence brightness, MC/s, million counts per second, of 54 nanojet devices measured on a single patterned type IIb diamond sample under an excitation power of ~0.9 mW. (**B**) Background-corrected second-order autocorrelation *g*^(2)^ (τ = 0) for the eight brightest devices. (**C**) Extracted saturation count rates for the selected devices. (**D**) Background suppression factor, defined as the ratio of coherent background collected through a nanojet to that measured on bare diamond *B*^Bare^/*B*^Nanojet^. (**E**) Excitation enhancement factor $E_{\text{eff}}$, defined as the local excitation field intensity enhancement at the targeted colour center(s) relative to the illumination field intensity.

Although the sample contains an inherently uncontrolled distribution of color centers and nitrogen concentration, a yield test still provides a meaningful measure of the robustness of the nanojet approach, which relies on stochastic coupling between individual color centers and the nanojet mode. We performed PL scans over a larger region of the sample containing more than 50 devices, correcting the height offset of each device individually to ensure optimal focus across the scan. The resulting brightness histogram, measured at an excitation power of 0.9 mW, is shown in Fig. 5A. The brightness of each device depends sensitively on how strongly the nanojet couples to the random spatial distribution of NV centers. We selected eight brightest nanojet devices out of this set and measured their background-subtracted autocorrelation statistics at around 0.9-mW excitation power, saturation behavior, background suppression ratio, and the excitation enhancement. These results are shown in Fig. 5 (B to E), respectively. Because the autocorrelation statistics are measured near the saturation power, coherent noise from background NV centers contributes significantly to the signal. The saturation counts indicate how efficiently individual NV centers are coupled out through the nanojet. The background-suppression ratio is inherently more sensitive to device-to-device variation, as it depends on how well the selected nanojet couples to its local NV environment compared with the randomly distributed background NVs on the surface. We also note a nonuniform distribution of background fluorescence even within small regions of the diamond sample. Overall though, it appears that at least 15% of the devices demonstrate a successful isolation of individual NV centers with the PNJs and enhanced collection of light emitted by the NVs. Higher-quality sample will likely to lead to an even better yield.

The optical performance of the device is quantified by the excitation enhancement, extracted by comparing the saturation power of the nanojet to that measured on bare diamond (Supplementary Materials section S2). Because the saturation power scales inversely with the local excitation intensity at the emitter, the ratio $P_{\text{sat}}^{\text{Bare}}/P_{\text{sat}}^{\text{Nanojet}}$ provides a measure of nanojet-induced excitation field enhancement. To obtain a quantitative estimate of the effective excitation enhancement $E_{\text{eff}}$, we combine calibrated measurements of the excitation beam profile with a confocal collection model that accounts for the partially saturated bulk NV ensemble in bare diamond. This model introduces an effective scaling factor $\alpha_{\text{eff}}={|E_{\text{eff},\text{diamond}}|}^{2}/{|E_{\text{peak},\text{air}}|}^{2}$, yielding

$$E_{\text{eff}}=\alpha_{\text{eff}}\frac{P_{\text{sat}}^{\text{Bare}}}{P_{\text{sat}}^{\text{Nanojet}}}$$

Here, α_eff_ maps the incident excitation power to the effective excitation intensity experienced by the emitters that dominate the detected signal. It captures (i) the nonuniform sampling of the Gaussian excitation profile imposed by the finite confocal collection mode—which preferentially weights emitters near the beam center (and, for shallow NVs, near the surface)—and can be expressed as a confocal-weighted effective intensity ${|E_{\text{eff},\text{diamond}}|}^{2}=\langle {|E_{\text{diamond}}\left(x,y,z,\lambda \right)|}^{2}\rangle$; and (ii) the reduction of the excitation field transmitted into bare diamond due to refraction at the air-diamond interface (Snell/Fresnel transmission). The enhancement ratio was determined by accounting for the experimental uncertainties in the measurement setup, and the values are shown in Fig. 5C (see Supplementary Materials section S2). The numbers are consistent in order of magnitude with the simulation results presented in Fig. 2A.

## DISCUSSION

We have demonstrated a monolithic, inverse-designed photon extractor carved directly into bulk diamond that exploits a PNJ to selectively excite and efficiently collect emission from individual NV centers. By combining near-field excitation confinement with engineered far-field radiation control, the device simultaneously enhances photon extraction and suppresses background PL from near-surface and randomly distributed defects, even in an uncontrolled type Ib HPHT diamond sample.

A central feature of this platform is its fully monolithic implementation based solely on air-diamond index contrast. The structures are fabricated using standard top-down lithography and reactive ion etching, without additional deposited layers, bonded optics, external microlenses, or separate near-field probes. This eliminates the need for substrate thinning, sacrificial layers, Faraday-cage or multistep etching, layer transfer, or pick-and-place alignment (*19*) and makes the approach compatible with large-area arrays patterned directly into bulk material.

PNJs offer several properties that are attractive for broadband PL collection from solid-state emitters. First, the near-field focusing mechanism provides strong spatial localization of the excitation field (*42*), enabling tighter excitation confinement than is typically achieved with purely lens-based approaches such as metasurfaces (*26*, *43*) or solid-immersion lenses. Because this behavior arises in a dielectric, nonresonant platform, it is intrinsically broadband and low loss, without requiring metallic components. Second, since the nanojet is formed in the near field, it can preferentially address a targeted emitter while reducing coupling to unwanted background channels, including substrate-guided radiation. Third, the nonresonant nature of nanojet formation supports broadband operation, making the approach naturally compatible with collecting emission across the phonon sideband of NV centers at room temperature.

Several previously reported photonic structures for NV centers are likewise monolithic, including pillar-based metasurfaces and metalens-type interfaces (*26*, *43*), as well as Fresnel- or zone plate–inspired solid-immersion optics (*44*). These devices generally improve fluorescence collection by shaping the emitted field for coupling into a desired free-space mode through conventional wavefront engineering. The closest prior work is (*29*), which also uses topology optimization to enhance photon extraction from diamond color centers. In contrast, the topology optimization in our work is formulated to directly generate a PNJ within the host material, so that this localized near-field feature becomes an explicit part of the device operation. This enables localized excitation and photon extraction to be addressed within the same monolithic diamond structure. Because the excitation volume is defined by the nanojet, the device can selectively address emitters within dense ensembles while also favoring upward-propagating free-space emission. In addition, the nanojet provides strong transverse confinement together with a comparatively extended axial high-intensity region, making the excitation less sensitive to uncertainty in emitter depth.

In contrast to approaches that physically isolate emitters through aggressive etching—such as nanowires (*11*, *22*), nanobeams, or ultrathin membranes (*10*)—as well as techniques developed for photon extraction from near-surface emitters (*45*), the nanojet architecture aims to provide optical isolation while maintaining a comparatively large emitter surface separation. By keeping the addressed emitter farther from etched interfaces, the design can reduce decoherence channels associated with paramagnetic surface spins and surface-induced electric field noise, thereby helping preserve the favorable coherence properties of bulk-like emitters (*46*).

Experimentally, the observed saturation count rate (*C*_sat_ ≈ 0.46 × 10^6^ cps) at low excitation power and clear antibunching [*g*^(2)^(0) ≈ 0.17] confirm that individual NV centers are selectively addressed, with effective suppression of background fluorescence. These results demonstrate that high-purity material and deterministic defect placement are not prerequisites for isolating single emitters when sufficiently strong near-field control is available. Future work will integrate deterministic NV implantation to improve device yield and enable on-demand single-photon sources.

Last, the inverse design framework presented here does not rely on any diamond-specific processing. It only assumes a high-index host that supports a nanojet and contains embedded optically active quantum emitters, making it broadly applicable beyond NV centers in diamond. The same principles can be extended to other diamond color centers, defects in SiC (*33*, *34*), rare-earth ions (*36*, *47*), optically active defects in silicon (*18*, *35*), and to fiber- or waveguide-integrated architectures for quantum network nodes.

In summary, free-form inverse design of monolithic PNJ extractors provides a scalable route to isolating and efficiently collecting emission from individual solid-state quantum emitters embedded in high–refractive index materials, unifying extreme near-field confinement with tailored far-field radiation control.

## MATERIALS AND METHODS

### Inverse design

The iterative optimization begins with continuous grayscale optimization of the design region, where the relative primitivity is weighted between that of diamond and that of air. The etched pattern is parametrized by a bounded field ρ(r)∈[0,1] defined on the device region Ω_d_ so $\varepsilon \left(r,\omega \right)=\varepsilon_{\text{air}}+\bar{\rho }\left(r\right)\left(\varepsilon_{\text{dia}}\left(\omega \right)-\varepsilon_{\text{air}}\right)$. A density filter (Helmholtz kernel of radius *r*_min_) and a smoothed Heaviside projection are applied to enforce a minimum feature size and near-binary layouts. We used the Lumerical FDTD engine to perform forward and adjoin simulations iteratively. The computational domain uses perfectly matched layers and conformal meshing; the mesh size is chosen to be ≤λ/(10*n*) everywhere in Ω_d_.

We maximize a scalar merit that jointly promotes nanojet focusing at the emitter and NA-limited broadband collection via reciprocity theorem

$$F\left(\rho \right)=\sum_{\omega_{k}\in \Lambda }I_{k}\;J_{\text{overlap}}\left(\rho ,\omega_{k}\right)$$

where $J_{\text{overlap}}\left(\rho ,\omega_{k}\right)$ denotes the mode overlap between the field radiated by an x-polarized dipole at the location of the targeted NV and a diffraction-limited Gaussian mode whose diameter corresponds to the Airy disk at the focal distance of the objective used in the measurement, evaluated at a height offset of Δ*z* = 2 μm and at frequency ω*_k_*. The spectral coefficients *I_k_* reproduce the NV fluorescence spectrum while also incorporating radiation near 532 nm to account for above-band excitation. Excitation and collection are treated symmetrically in accordance with the reciprocity theorem. In practice, only a limited number of spectral components are required, as the adjoint method inherently converges toward a broadband solution. Notably, because the same objective is used for both excitation and collection, maximizing the dipole emission at the location of the selected NV simultaneously optimizes the excitation channel, in accordance with the reciprocity theorem.

Gradients are computed with one adjoint solve per term/frequency and incoherently summed up over Λ; designs are updated with the L-BFGS-B algorithm using continuation on the projection slope and weights. Convergence is declared by stagnation of *F*, gradient-norm reduction, and near-binary metrics; final layouts are resimulated on a refined mesh.

### Sample fabrication

The nanofabrication process begins with a single-crystal HPHT yellow diamond (Element Six), sequentially cleaned with acetone, isopropyl alcohol (IPA), and mild oxygen plasma ashing to remove surface contaminants. The diamond is secured in a custom silicon pocket using a thin poly(methyl methacrylate) layer and then baked at 180°C for 10 min. After cooling, a 1:1 diluted ZEP 520A (diluted with Anisol) resist is spin-coated at 3000 rpm for 60 s, followed by soft baking at 180°C for 5 min. To minimize charging during electron beam exposure, a conductive Electra92 polymer layer is applied via spin coating.

EBL is performed using a JEOL EBL 6300FS system with proximity effect correction via BGenISys BEAMER-TRACER software package. After exposure, the Electra92 layer is removed in deionized (DI) water, and after air drying, the sample is developed in ZED-N50 for 90 s. A 30-nm titanium hard mask is deposited via electron beam evaporation (angstrom deposition system). The lift-off process involves soaking in Remover PG solvent and ultrasonic agitation, followed by DI water and IPA rinsing with nitrogen drying. Etching is conducted using an Oxford Plasmalab system 100 ICP380 RIE system under optimized conditions: 700-W ICP (inductively coupled plasma) power, 100-W RF power, 10-mtorr chamber pressure, 25°C substrate temperature, and 30 sccm oxygen gas flow for 8 min. Last, the titanium mask is removed with a 1:5 diluted hydrofluoric acid solution, followed by thorough DI water rinsing and nitrogen-assisted drying.

### Measurement procedure

The scanning microscope setup (Fig. 3B) features an infinity-corrected 100× Olympus M Plan ApoN100×/0.95 objective with NA = 0.95, alongside confocal lenses with anti-reflection coating (Thorlabs, AC254-200-AB and AC254-100-AB) and with 200- and 100-mm focal lengths, respectively. A pinhole (marked SF in Fig. 3B) acts as a spatial filter removing stray light. A 40-nm-wide bandpass filter (marked as BP) centered at 520 nm (Thorlabs, FBH520-40) removes background noise in the florescence band of the NV centers. This background noise arises due to Brillouin/Raman scatteringof the green excitation laser in the input fiber. A set of additional optical filters consisting of a 650-nm cut-off long-pass filter (Thorlabs, FELH0650), a 550-nm cut-off longpass filter (Thorlabs, FELH0550), a 950-nm cut-off short-pass filter (Thorlabs, FES0950), and a 17-nm-wide notch filter centered at 533 nm (Thorlabs, NF533-17) is used to eliminate unwanted background and excitation light in the collection path. These filters are collectively marked as LP in Fig. 3B.

A two-axis galvo mirror (Thorlabs, GVS002) enables scanning via 4f relay optics, while a piezo stage controls height measurement and coarse scanning. The sample is excited with a 532nm continuous-wave laser (Coherent, Compass 315M-100 CW) that is coupled into a single-mode fiber to achieve a Gaussian spatial beam profile. For lifetime measurements, we used a 520-nmgain-switched laser (Thorlabs, GSL52A), set to 130-ps pulse width and low repetition rate.

PL emission is fiber-collected via an 10× objective (Olympus Plan N 10×/0.25CY). Here, confocality is achieved via fiber coupling. The polarization control module, marked as POL in Fig. 3B, consists of a quarter-wave plate, a half-wave plate, and a polarizer, and is used to perform polarimetric tomography. The fluorescence spectrum of the emission is measured using a home-built Czerny Turner spectrometer comprising a 150 ln/mm grating and a high-sensitivity imaging camera (ZW Optical, ASI2600M). For ODMR detection, the sample is directly placed on a microstrip line fed by a microwave synthesizer.

Photon detection is carried out using single-photon counting modules (Excellitas, 850-14-FC) and superconducting-nanowire single-photon detectors (ID Quantique, ID281) for 2D scans, saturation curves, lifetime measurements, and ODMR data acquisition. The photon detection events are recorded as counts with an electronic time-tagger (ID Quantique, ID900). A 50-50 fiber-beam splitter (Thorlabs, PN670R5A2) enables beam splitting in the HBT configuration for second-order correlation measurements. A custom-built software system locks onto the target spot and compensates for sample vibrations, ensuring stability in long-duration measurements.
