# Supplementary material for: Probing individual quantum emitters in bulk semiconductors via photonic nanojets
Source: Sci Adv. 2026 May 20;12(21):eaea5936. doi: 10.1126/sciadv.aea5936 (PMC13189122; doi:10.1126/sciadv.aea5936)
Supplement: Supplementary file 1 — Supplementary Text Figs. S1 to S11 [file sciadv.aea5936_sm.pdf]

Science Advances
AAAS

Supplementary Materials for

Probing individual quantum emitters in bulk semiconductors via photonic nanojets

Behrooz Semnani *et al.*

Corresponding author: Behrooz Semnani, bsemnani@uwaterloo.ca; Michal Bajcsy, mbajcsy@uwaterloo.ca

Sci. Adv. **12**, eaea5936 (2026)
DOI: 10.1126/sciadv.aea5936

This PDF file includes:

Supplementary Text
Figs. S1 to S11

1 Photonic Nanojet Design

1.1 Initial Geometry Selection

Since adjoint inverse topology optimization is a gradient-based technique, initial conditions play a critical role in the design process. To ensure a nanojet excitation, we carefully define an initial geometry capable of generating a nanojet. This approach has proven highly effective in achieving nanojet excitation in the final optimized structure. The initial geometry selection follows the argument in [38], where edge formation is analyzed based on Huygens' principle. Edge jets can condense into a bright spot at the designated location. The geometric argument and critical conditions for edge jet formation is shown in the main body of manuscript, while simulation results illustrating nanojet formation at 532 nm illumination for the initial geometry are shown below.

1.2 Inverse Adjoint Topology Optimization

We parameterize the etched pattern by a bounded field $\rho(\mathbf{r}) \in [0, 1]$ defined on the device region Ω_d. After applying a *density filter* (Helmholtz kernel of radius r_{min}) and a *smoothed Heaviside projection* (slope parameter β) to enforce a minimum feature size and near-binary layouts, the permittivity reads

$$\varepsilon(\mathbf{r}, \omega) = \varepsilon_{\text{air}} + \bar{\rho}(\mathbf{r})\big(\varepsilon_{\text{dia}}(\omega) - \varepsilon_{\text{air}}\big), \tag{1}$$

with $\bar{\rho}$ the filtered–projected design. The computational domain uses perfectly matched layers (PML) and conformal meshing; the mesh size is chosen to be $\leq \lambda/(10n)$ everywhere in Ω_d. The simulation setup is shown in Fig. S2.

Discretization and boundary conditions

PML thickness $\geq 1.0\,\lambda/n$. Mesh resolution $\leq \lambda/(10n)$ in Ω_d and $\leq \lambda/(8n)$ near high-curvature features. Simulations are verified for NA truncation by enlarging S_z and by near-to-far-field consistency checks. Reported efficiencies include objective transmission unless stated otherwise.

1.3 Broadband Optical Response of the Inverse Design Structure

The photonic nanojet provides non-resonant field confinement in the bulk. We demonstrate that this field enhancement mechanism is broadband, enabling precise tailoring of the radiation pattern across a wide spectral range within the NV fluorescence bandwidth at room temperature.

1.4 Purcell Enhancement

To assess the effect of near-field coupling between the emitter and the nanojet extractor on the emission decay rate, we model the colour centres as classical electric dipoles located at experimentally relevant depths. Using FDTD simulations performed in Ansys Lumerical, we compute the total radiated power in the presence of the nanojet, which is proportional to the local density of optical states. By normalizing this value to the total radiated power of an identical dipole in bulk diamond ($n = 2.41$) without the nanojet, we obtain the Purcell factor F_P. Figure S4**a** shows the wavelength dependence of F_P for dipoles oriented along the x and y directions, respectively.

However, the parameter most directly comparable to the experimentally measured Purcell enhancement is the spectrally weighted Purcell factor, defined as

$$F_P^{(\mathrm{eff})} = \frac{\int S(\lambda)\, F_P(\lambda)\, d\lambda}{\int S(\lambda)\, d\lambda}, \tag{2}$$

where $S(\lambda)$ is the experimentally measured emission spectrum of the colour centres. The spectra used for this weighting, corresponding to emitters at different depths beneath the structure, are shown in Fig. S4**b**.

2 Excitation Enhancement Analysis

The topology-optimized photonic nanojet device provides excitation enhancement, as demonstrated by the numerical simulation results presented in the main text. This section focuses on the experimental confirmation of this excitation enhancement, followed by data analysis based on the actual measured experimental parameters.

To estimate the excitation enhancement at the wavelength of 532nm, it is first important to note that the enhancement depends sensitively on the illumination beam profile used to probe the structure. Because the same confocal microscope is used for both excitation and collection, accurately determining the enhancement requires an initial measurement of the beam profile of the green-laser.

A systematic way to estimate the excitation enhancement is to compare the saturation power levels for emission through the nanojet device with those measured on bare diamond, where ensembles of NV centres are illuminated by a focused beam. As discussed in the main text, the nanojet bright spot saturates at low excitation powers, whereas the background exhibits a linear response. However, accurately determining the enhancement requires a more dedicated analysis that accounts for the characteristics of the confocal setup used to collect the PL. To estimate the excitation enhancement provided by the nanojet device, we follow the steps below:

1. **Measure the beam profile of the 532nm laser used to probe the structure.**

2. **Record the saturation map of the nanojet bright spot**, confirming its low-power saturation behaviour ($P_{\text{sat}}^{(\text{Jet})}$).

3. **Measure the saturation power on bare diamond**, with the beam focused directly on the surface, in contrast to the nanojet measurement where the beam waist is initially offset. The saturation hapens in much larger power range ($P_{\text{sat}}^{(\text{bare})}$)

4. **Model the PL collected from ensembles of emitters in bare diamond**, noting that the saturation behaviour is dominated by how a Gaussian beam excites the emitters and how their collective fluorescence is captured. This requires knowledge of the confocal setup, including the effective collection-spot size on the sample and the contribution of bare diamond to the detected PL. This analysis yields a normalization factor that accounts for the fact that the confocal system collects fluorescence from an ensemble of colour centres rather than from a single NV centre. The normalization factor, denoted by $\alpha_{\text{eff}} = |E_{\text{eff,diamond}}|^2 / |E_{\text{peak,air}}|^2$, is defined as the effective excitation intensity inside the diamond relative to that in air, taking into account the fraction of PL that is effectively collected by the confocal setup.

5. **Relate the PL contribution from bare diamond to the laser intensity in air**, allowing the comparative saturation powers of the nanojet device and bare diamond to be used to estimate the excitation enhancement.

$$\mathcal{E}_{\text{eff}} \approx \alpha_{\text{eff}} \frac{P_{\text{sat}}^{\text{Bare}}}{P_{\text{sat}}^{\text{Nanojet}}} \tag{3}$$

It is important to note that the enhancement is defined as the excitation-field enhancement relative to a Gaussian beam within the Rayleigh range of the illumination spot in air. This provides the closest experimentally accessible parameter to the simulated enhancement values presented in the main text.

2.1 Field simulation using measured beam profile

We first characterized the beam profile of the 532 nm laser. To do this, we measured the reflected signal from a calibration sample with known dimensions. The resulting beam profile within the Rayleigh range of the Gaussian focus is shown in Fig.S5 (a). Because the back focal plane of the objective is not fully filled, the effective divergence angle is approximately 29°, corresponding to a beam-waist diameter of $\sim 0.4\,\mu$m. To estimate both the beam size and the divergence angle, we use the following method.

The beam profile has been captured in various heights. From each post-processed image $I(x, y)$ (after background subtraction), we compute the intensity-weighted centroid

$$x_0 = \frac{\sum Ix}{\sum I}, \qquad y_0 = \frac{\sum Iy}{\sum I}, \tag{4}$$

and the 2×2 covariance matrix

$$\mathbf{C} = \begin{pmatrix} \langle (x - x_0)^2 \rangle & \langle (x - x_0)(y - y_0) \rangle \\ \langle (x - x_0)(y - y_0) \rangle & \langle (y - y_0)^2 \rangle \end{pmatrix}. \tag{5}$$

Diagonalizing $\mathbf{C}$ yields eigenvalues σ_1^2, σ_2^2 and eigenvectors defining the principal axes. The spot rotation angle is the angle of the major-axis eigenvector $\mathbf{v}$,

$$\theta = \mathrm{atan2}(v_y, v_x), \tag{6}$$

and the corresponding $1/e^2$ radii are

$$w_1 = 2\sigma_1, \qquad w_2 = 2\sigma_2. \tag{7}$$

This procedure is well behaved for incoherent broadband mixtures because second moments commute with incoherent intensity addition.

We use the measured beam profile to simulate the excitation of the structure under actual experimental conditions. The results are shown in Fig. S5(b) and (c) for bare diamond and nanojet excitation, respectively. To achieve saturation on bare diamond, the beam is brought sufficiently close to the sample surface to maximize the local field intensity and drive the diamond into saturation at lower power. This experimental condition is reproduced in the simulation shown in Fig. S5(b). In contrast, for nanojet excitation, as described in the main text, an appropriate z offset is applied to minimize background fluorescence while selectively exciting the nanojet mode.

2.2 Saturation Measurements

The saturation curves of both the bare diamond and the nanojet device were measured using high-power excitation. In particular, we used the pump laser of a continuous Ti:sapphire laser to arbitrarily increase the available power and observe the saturation behaviour on a bare region of the diamond sample located near the selected nanojet devices. It was also necessary to ensure that the beam was sufficiently well focused on the sample so that the local intensity was high enough to drive the NV centres into saturation. From the measurements, we confirmed that the sample was positioned within the Rayleigh range of the excitation beam, which is approximately 1.2 μm, and this also sets the uncertainty in the intensity profile. Figure S6 shows the extracted saturation power for the bare diamond under a tightly focused beam, as well as the saturation behaviour of the nanojet device measured with an appropriate z-offset.

The bare-diamond PL is collected from multiple locations to ensure that variations and measurement uncertainties are properly accounted for. The red error bars in Fig. S6(a) represent the variation in the PL signal from bare diamond for different input powers. The yellow shaded region shows the fitted saturation function, which yields a saturation power of $P_{\mathrm{sat}}^{\mathrm{Bare}} = 63.0\,\mathrm{mW} \pm 4\,\mathrm{mW}$. In contrast, the saturation power measured for the selected nanojet device is much smaller, $P_{\mathrm{sat}}^{\mathrm{Jet}} = 0.93\,\mathrm{mW}$, confirming the strong ability of

the device to tightly confine light.

2.3 Modeling Illumination and Confocal Collection from Bare Diamond

We model photoluminescence (PL) from near-surface NV centers in diamond under Gaussian excitation launched in air and collected through a confocal microscope equipped with a single-mode fiber (SMF). The detected signal is computed by combining (i) a plane-wave (angular spectrum) description of excitation transmission through the air–diamond interface, (ii) incoherent broadband confocal collection modeled as an intensity weighting function spectrally averaged over the NV emission band, and (iii) a local saturation law for each emitter. Unless stated otherwise, the model is scalar; thus, polarization effects enter through Fresnel transmission coefficients for s and p polarizations or a simple mixture of the two.

Incident Gaussian field in air. At a reference plane in air (taken as the waist plane), we define a scalar field

$$E_{\mathrm{air}}(x, y; z = z_w) = E_0 \exp\left(-\frac{x^2 + y^2}{w_0^2}\right), \tag{8}$$

so that the intensity has $1/e^2$ radius w_0:

$$I_{\mathrm{air}}(x, y; z) = \frac{1}{2} n_{\mathrm{air}} \varepsilon_0 c \, |E_{\mathrm{air}}(x, y; z)|^2. \tag{9}$$

The amplitude E_0 is chosen such that the total power in the waist plane equals a reference power P_0:

$$P_0 = \iint I_{\mathrm{air}}(x, y; z = z_w) \, dx \, dy. \tag{10}$$

Angular spectrum propagation to the interface. We compute the angular spectrum at z as

$$A_{\mathrm{air}}(k_x, k_y; z) = \mathcal{F}\{E_{\mathrm{air}}(x, y; z)\}, \tag{11}$$

and propagate over distance Δz in a medium of refractive index n using

$$A(k_x, k_y; z + \Delta z) = A(k_x, k_y; z) \exp(ik_z \Delta z), \tag{12}$$

with longitudinal wavevector component

$$k_z = \sqrt{(nk_0)^2 - k_x^2 - k_y^2}, \qquad k_0 = \frac{2\pi}{\lambda_{\text{exc}}}. \tag{13}$$

An inverse Fourier transform yields the field at the air side of the interface ($z = 0^-$).

Plane-wave transmission across the air–diamond interface. For each transverse wavevector component (k_x, k_y), transverse momentum is conserved at the interface while the longitudinal component changes from

$$k_{z,1} = \sqrt{(n_{\text{air}}k_0)^2 - k_x^2 - k_y^2} \quad \rightarrow \quad k_{z,2} = \sqrt{(n_{\text{d}}k_0)^2 - k_x^2 - k_y^2}, \tag{14}$$

where n_{d} is the diamond refractive index at λ_{exc}. Defining

$$\cos\theta_1 = \frac{k_{z,1}}{n_{\text{air}}k_0}, \qquad \cos\theta_2 = \frac{k_{z,2}}{n_{\text{d}}k_0}, \tag{15}$$

the Fresnel field transmission coefficients are

$$t_s = \frac{2n_{\text{air}}\cos\theta_1}{n_{\text{air}}\cos\theta_1 + n_{\text{d}}\cos\theta_2}, \tag{16}$$

$$t_p = \frac{2n_{\text{air}}\cos\theta_1}{n_{\text{d}}\cos\theta_1 + n_{\text{air}}\cos\theta_2}. \tag{17}$$

In the scalar approximation, we use $t = t_s$, $t = t_p$, or a polarization-averaged coefficient $t = (t_s + t_p)/2$ to represent unknown polarization mixture.

The transmitted angular spectrum at $z = 0^+$ is then

$$A_\mathrm{d}(k_x, k_y; 0^+) = A_\mathrm{air}(k_x, k_y; 0^-)\, t(k_x, k_y). \tag{18}$$

Propagation inside diamond and excitation intensity. Within diamond, the angular spectrum propagates as

$$A_\mathrm{d}(k_x, k_y; z) = A_\mathrm{d}(k_x, k_y; 0^+) \exp(ik_{z,2}z), \tag{19}$$

and the field is reconstructed by inverse Fourier transform,

$$E_\mathrm{d}(x, y; z) = \mathcal{F}^{-1}\{A_\mathrm{d}(k_x, k_y; z)\}. \tag{20}$$

The excitation intensity in diamond is

$$I_\mathrm{d}(x, y; z) = \frac{1}{2} n_\mathrm{d} \varepsilon_0 c \, |E_\mathrm{d}(x, y; z)|^2. \tag{21}$$

With the normalization of Eq. (10), we obtain $I_\mathrm{d}(x, y; z) = P\, I_\mathrm{unit}(x, y; z)$, where I_unit is the computed intensity per watt.

Incident reference intensity at the surface. To compare the excitation in diamond against the incident field in air, we also compute the incident intensity at the surface plane for the *diamond-absent* case:

$$I_\mathrm{air}(x, y; z = 0) = \frac{1}{2} n_\mathrm{air} \varepsilon_0 c \, |E_\mathrm{air}(x, y; 0)|^2, \tag{22}$$

and define the peak incident intensity

$$I_\mathrm{peak,air} = \max_{x,y} I_\mathrm{air}(x, y; 0). \tag{23}$$

Confocal Collection with Single-Mode Fiber for Incoherent Broadband PL

Incoherent intensity weighting. NV PL is spontaneous emission and is therefore treated as incoherent; collection is modeled by an *intensity* weighting function rather than coherent mode overlap. We approximate the confocal+SMF detection weighting as a separable Gaussian in the sample:

$$W(r, z; \lambda) = \exp\left(-\frac{2r^2}{w_{\text{det}}(\lambda)^2}\right) \exp\left(-\frac{2(z - z_f)^2}{z_{\text{det}}(\lambda)^2}\right), \tag{24}$$

where $r = \sqrt{x^2 + y^2}$, z_f is the detection focus depth, and w_{det}, z_{det} are effective lateral and axial detection radii in the sample.

Broadband spectral averaging. The detected signal integrates over a wide emission band, and the confocal acceptance varies with wavelength. We therefore compute a spectrally averaged detection weighting:

$$\overline{W}(r, z) = \int w(\lambda)\, W(r, z; \lambda)\, d\lambda, \tag{25}$$

with normalized weights

$$w(\lambda) = \frac{S_{\text{NV}}(\lambda)\, T_{\text{optics}}(\lambda)\, \text{QE}(\lambda)}{\int S_{\text{NV}}(\lambda)\, T_{\text{optics}}(\lambda)\, \text{QE}(\lambda)\, d\lambda}. \tag{26}$$

To capture wavelength dependence of the detection PSF we use a power-law scaling

$$w_{\text{det}}(\lambda) = w_{\text{det}}(\lambda_0) \left(\frac{\lambda}{\lambda_0}\right)^{\alpha_{\text{lat}}}, \qquad z_{\text{det}}(\lambda) = z_{\text{det}}(\lambda_0) \left(\frac{\lambda}{\lambda_0}\right)^{\alpha_{\text{ax}}}, \tag{27}$$

with $\alpha_{\text{lat}} \approx \alpha_{\text{ax}} \approx 1$ as a starting point.

Near-Surface NV Distribution and Saturation Law

Depth distribution. We restrict the integration to a near-surface slab $0 \le z \le z_{\max}$ and optionally include a depth-dependent NV density $\rho(z)$ (e.g. an exponential implantation profile). The effective weighting is $\overline{W}(x, y, z)\rho(z)$.

Saturable excitation dependence. At each position the normalized emission is modeled by

$$s(I) = \frac{I}{I + I_{\text{sat}}}, \tag{28}$$

where I_{sat} is the local saturation intensity for a single NV (or effective ensemble average).

Detected Bulk PL Signal

The detected bulk PL signal at excitation power P is computed as

$$S(P) \propto \iiint_{z\in[0,z_{\max}]} \overline{W}(x, y, z)\, \rho(z)\, \frac{P\, I_{\text{unit}}(x, y, z)}{P\, I_{\text{unit}}(x, y, z) + I_{\text{sat}}}\, dV, \tag{29}$$

and is reported in normalized form:

$$\frac{S(P)}{S(\infty)} = \frac{\iiint \overline{W}\rho\, \frac{PI_{\text{unit}}}{PI_{\text{unit}}+I_{\text{sat}}}\, dV}{\iiint \overline{W}\rho\, dV}. \tag{30}$$

This normalization removes unknown proportionality factors such as absolute NV density and overall detection gain.

Single-NV Comparison and Confined-Mode Enhancement

For a single NV at position $\mathbf{r}_{\text{NV}}$, the local excitation intensity from the air-illuminated field is $I_{\text{loc}}(P) = P\, I_{\text{unit}}(\mathbf{r}_{\text{NV}})$. To represent excitation via a confined mode, we introduce an

intensity enhancement factor FE (default $FE = 1$):

$$I_{\text{mode}}(P) = FE \cdot I_{\text{loc}}(P). \tag{31}$$

The normalized single-emitter saturation curve is then

$$\frac{S_{\text{single}}(P)}{S_{\text{single}}(\infty)} = \frac{I_{\text{mode}}(P)}{I_{\text{mode}}(P) + I_{\text{sat}}}. \tag{32}$$

If a field (amplitude) enhancement $|E|$ is known instead, the corresponding intensity enhancement is $FE = |E|^2$.

Effective Excitation Intensity and the Impact of w_{det}

To quantify how strongly the confocal measurement probes the excitation distribution in diamond, we define a linear-regime effective intensity:

$$I_{\text{eff,diamond}} = \frac{\iiint \overline{W}(x,y,z)\rho(z)\, I_{\text{unit}}(x,y,z)\, dV}{\iiint \overline{W}(x,y,z)\rho(z)\, dV}. \tag{33}$$

This can be compared to $I_{\text{peak,air}}$ defined above (diamond absent) or to a laterally weighted incident reference intensity at $z = 0$ using the projected weighting $W_{xy}(x,y) = \int \overline{W}(x,y,z)\rho(z)\, dz$.

The parameters w_{det} and z_{det} govern which spatial regions contribute to the detected PL. Decreasing w_{det} emphasizes the beam center (higher intensity), increasing $I_{\text{eff,diamond}}$ and making saturation appear more pronounced. Increasing w_{det} includes more low-intensity tails, which adds an approximately linear component and shifts the apparent saturation to higher power. Similarly, reducing z_{det} increases axial sectioning and makes the signal more sensitive to near-surface intensity gradients and to the depth distribution $\rho(z)$.

Uncertainty Propagation

Uncertainties in w_0, z_w, polarization mixture, n_d, and the confocal parameters (w_det, z_det, z_f), as well as the depth profile $\rho(z)$, can significantly affect $I_\mathrm{eff,diamond}/I_\mathrm{peak,air}$ and the predicted saturation curves. We propagate such uncertainties via Monte-Carlo sampling of these parameters and recomputation of Eqs. (30) and (33), reporting mean $\pm$ standard deviation or percentile intervals as error bars.

Since the dependence on the excitation beam size and the effective detection beam size on the sample is particularly decisive for the contribution and saturation behaviour of the bare diamond, we measured both the excitation spot size and the detection spot size. The detection beam size was obtained by injecting a red laser (637 nm) into the single-mode fibre of the collection path and imaging the resulting spot on the camera. The results are shown in Fig. S7(a). We measure an excitation-beam diameter of $W_\mathrm{exc} \approx 0.4\,\mu$m, corresponding to illumination at a height offset of $\sim 1\,\mu$m, and a detection-beam diameter of $W_\mathrm{det} \approx 0.9\,\mu\mathrm{m} \pm 0.2\,\mu$m at a height offset of $\sim 2{-}3\,\mu$m. These uncertainties are incorporated into the Monte Carlo simulations.

The effective scaling factor, defined below, is then estimated as

$$\alpha_\mathrm{eff} = \frac{|E_\mathrm{eff,diamond}|^2}{|E_\mathrm{peak,air}|^2} = \frac{I_\mathrm{eff,diamond}}{n_\mathrm{diamond} I_\mathrm{peak,air}} \approx 0.125 \pm 0.014 \tag{34}$$

The scaling factor has two main contributions: first, the effective collection of PL from the distribution of NV centres excited across the Gaussian beam used to probe the sample. This contribution, shown in Fig. S7(c), explains the difference between the saturation behaviour of an ensemble and that of a single NV centre. The second contribution arises from the reduction in excitation intensity inside the diamond due to Fresnel reflection at the air–diamond interface

Finally, we use the saturation powers estimated from Fig. S6 together with the factor α_eff to estimate the enhancement of the excitation field intensity with respect to the field within

the Rayleigh range of the probing microscope. We also present the raw enhancement factor, defined simply as the ratio of the nanojet signal to that of bare diamond, even when the excitation beam is tightly focused on the sample. The results are shown in Fig. S8(a) and (b), respectively.

$$\mathcal{E}_{\text{raw}} = \frac{P_{\text{sat}}^{(\text{Bare})}}{P_{\text{sat}}^{(\text{Jet})}} \tag{35}$$

$$\mathcal{E}_{\text{eff}} = \alpha_{\text{eff}} \mathcal{E}_{\text{raw}} \tag{36}$$

3 Simulation of Second-Order Correlation Measurement from an NV Center

Here, a model for simulating the emitted photon statistics from a single NV center is presented. Fig. S9.a illustrates the energy level structure of a negatively charged NV center and its corresponding decay rates at room temperature. The decay rate γ shown in the figure is estimated based on our measured lifetime of NV center fluorescence, which is found to be $\tau = 9\,\text{ns}$. The other decay rates correspond to an NV center in bare diamond [26]. When excited with unpolarized light, this structure can be represented by a simplified three-level model as shown in Fig. S9.b with equivalent decay rates Γ_1 to Γ_3 where $\Gamma_1 = \gamma$ and $\Gamma_3 = D_0 + D_1$. To calculate Γ_2, one needs to find the ratio of the population of excited states with $m_s = 0$ and $m_s = \pm 1$ at steady state. To do that, we calculate the steady state solution of the following recursive equations:

$$\begin{aligned} \rho_{22}(i+1) &= \tfrac{\gamma}{\gamma+S_0}\rho_{22}(i) + \tfrac{D_0}{D_0+D_1}\left[\tfrac{S_0}{\gamma+S_0}\rho_{22}(i) + \tfrac{S_1}{\gamma+S_1}\rho_{44}(i)\right] \\ \rho_{44}(i+1) &= \tfrac{\gamma}{\gamma+S_1}\rho_{44}(i) + \tfrac{D_1}{D_0+D_1}\left[\tfrac{S_0}{\gamma+S_0}\rho_{22}(i) + \tfrac{S_1}{\gamma+S_1}\rho_{44}(i)\right] \end{aligned} \tag{37}$$

where $\rho_{22}(i)$ and $\rho_{44}(i)$ are populations of states $|2\rangle$ to $|4\rangle$ after i thexcitation and de-

excitation. After finding the steady state solutions ρ_{22} and ρ_{44}, the below equation can be used to calculate the effective decay rate Γ_2:

$$\Gamma_2 = \frac{S_0}{1 + \rho_{44}/\rho_{22}} + \frac{S_1}{1 + \rho_{22}/\rho_{44}} \tag{38}$$

Starting from an initial condition $\rho_{22}(0) = 0$, and $\rho_{44}(0) = 1$, Fig. S10 shows how the ground state populations and the effective decay rate Γ_2 change as a function of number of excitation and de-excitation cycles. According to these results, at steady state we have $\Gamma_2 = 17.1\,\mathrm{MHz}$.

The total nonradiative decay rate of the model shown in Fig.S9b is $\Gamma_2\Gamma_3/(\Gamma_2 + \Gamma_3)$. Considering the time needed for excitation of the emitter and the fact that not all decays are radiative, the photon rate of the collected fluorescence $\bar{n}_{collected}$ is derived as follows:

$$\bar{n}_{collected} = \frac{\eta_{\mathrm{col}}\Gamma_1}{\frac{\Gamma_2+2\Gamma_3}{\Gamma_3} + E_{\mathrm{photon}}\frac{\Gamma_1+\Gamma_2}{\eta_{\mathrm{exc}}P}} \tag{39}$$

In the above equation, P represents the power of the excitation laser, E_{photon} is the energy of each individual exciting photon, η_{col} denotes the collection efficiency of the emitted fluorescence photons, and η_{exc} represents the excitation efficiency, which indicates the ratio of excitation photons incident on the NV center's cross-sectional area that can potentially excite the NV center. The estimated values for η_{col} and η_{exc} based on the measured count rates at different excitation powers are 0.012 and 4.6×10^{-9}, respectively.

If the emitter is in ground state at $t = 0$ when the laser pulse is turned on, the probability distribution of the time it needs to wait to get excited again will be:

$$p_{exc}(t) = \bar{n}\exp(-\bar{n}t) \tag{40}$$

where $\bar{n} = \eta_{\mathrm{exc}}P/E_{\mathrm{photon}}$ is the arrival rate of excitation photons which can potentially

interact with the NV center. In addition, the probability distribution of an initially excited emitter decaying at time t is:

$$p_{decay1}(t) = \Gamma_1 \exp(-\Gamma_1 t) \qquad p_{decay2}(t) = \frac{\Gamma_2\Gamma_3}{\Gamma_2 - \Gamma_3}\left(\exp(-\Gamma_3 t) - \exp(-\Gamma_2 t)\right) \tag{41}$$

where $p_{decay1}(t)$ and $p_{decay2}(t)$ correspond to the emitter decaying through each of the radiative or non-radiative channels, respectively. Denoting the branching ratio to the non-radiative channel by $r_b = \Gamma_2/\Gamma_1$, the decay of the emitter results to emission of a photon with probability $1/(1+r_b)$. Combining equations (4) and (5), the probability distribution of the time difference between two consecutive decays is obtained as follows:

$$p_{r(nr)}(t) = \int_0^t p_{exc}(\tau)\, p_{decay1(2)}(t-\tau)\, d\tau \tag{42}$$

where $p_r(t)$ and $p_{nr}(t)$ corresponds to the cases where the second decay is a radiative or a non-radiative decay, respectively. Substituting equations (4), (5) into (6), one obtains:

$$\begin{array}{l} p_r(t) = \frac{\bar{n}\Gamma_1}{(\Gamma_1-\bar{n})}\left[\exp(-\bar{n}t) - \exp(-\Gamma_1 t)\right] \\ p_{nr}(t) = \frac{\bar{n}\Gamma_2\Gamma_3}{\Gamma_2-\Gamma_3}\left[\frac{\exp(-\bar{n}t)-\exp(-\Gamma_3 t)}{\Gamma_3-\bar{n}} - \frac{\exp(-\bar{n}t)-\exp(-\Gamma_2 t)}{\Gamma_2-\bar{n}}\right] \end{array} \tag{43}$$

Giving the following cumulative distribution functions (CDFs):

$$\begin{array}{l} P_r(t) = 1 - \frac{\Gamma_1\exp(-\bar{n}t)-\bar{n}\exp(-\Gamma_1 t)}{\Gamma_1-\bar{n}} \\ P_{nr}(t) = 1 - \frac{1}{\Gamma_2-\Gamma_3}\left[\Gamma_2\frac{\Gamma_3\exp(-\bar{n}t)-\bar{n}\exp(-\Gamma_3 t)}{\Gamma_3-\bar{n}} - \Gamma_3\frac{\Gamma_2\exp(-\bar{n}t)-\bar{n}\exp(-\Gamma_2 t)}{\Gamma_2-\bar{n}}\right] \end{array} \tag{44}$$

Using equation (8) and adopting a random-walk perspective, one can generate a train of photon emission times, from which the simulated second-order correlation function (denoted as $g_s^{(2)}(\tau)$) can be obtained. To do that, at each step two random numbers r_1 and r_2 in the $[0,1]$ interval are generated. First, by comparing r_1 with the photon emission probability $1/(1+r_b)$ the radiative or non-radiative type of the decay is determined. Then,

by equating r_2 with the corresponding CDF, the time of the decay is determined. If the decay is radiative, one photon is radiated, and its radiation time is stored. Repeating this procedure, gives the emission time train and consequently the second-order correlation of the emitted fluorescence. To account for the effect of background illumination, we model it as an uncorrelated noise source with a count rate equal to the measured background count rate. The emission time series for the noise is then combined with that of a single NV center, resulting in a single time series.

Figure S5 shows both the measured and simulated second-order correlation results for three different excitation laser power values: $P = 0.3\,\mathrm{mW}$, $P = 0.6\,\mathrm{mW}$, and $P = 1.8\,\mathrm{mW}$. The bunching effect occurring around the central dip at $\tau = 0$ is a result of the existence of the non-radiative decay path and metastable state. The branching ratio of 25% means that on average from each five de-excitations one occurs through the non-radiative path. Furthermore, because of the long lifetime of the metastable state, it takes a long time for the emitter to decay through this path. Therefore, there are long time intervals of no photon emission between bunches of consequent photon emissions which are apart in time on average approximately by $1/\Gamma_1$.

4 Background Subtraction in $g^{(2)}$ Data

We measured the background contribution from the PL maps presented in the manuscript. 2D PL scans were performed at each power level, extracting the lowest count rate across the device (typically ∼500 nm apart from the nanojet spot) . As shown below, subtracting background counts establishes an upper limit for autocorrelation statistics. The brightest spot, corresponding to nanojet excitation, consists of both the background floor (B) and emission from NV centers within the nanojet mode (S).

Background correction of the cross-correlation data in Fig. S11 was performed using

the following equation [48,49]:

$$g^{(2)}(\tau) = \frac{1}{\rho^2}\left[g_m^{(2)}(\tau) + \rho^2 - 1\right] \tag{45}$$

Here $g_m^{(2)}(\tau)$ is the measured second-order correlation statistics and ρ is the ratio of background-corrected signal to total signal i.e. $\rho = S/(S+B)$. Different ρ values are expected for different power levels. We measured the ρ values of 0.47, 0.43 and 0.35 for the excitation powers of 0.3mW, 0.6mW and 1.8 mW respectively. The results are presented in Fig.4 e-g in the main body of manuscript.

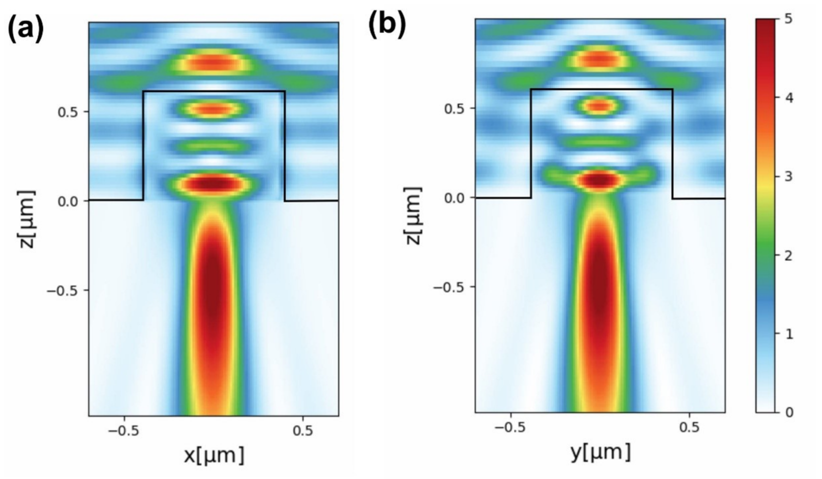

Figure S1: Initial geometry selection for photonic nanojet generation under 532 nm laser illumination. Field intensity distribution in (a) xz-plane and (b) yz-plane. The step-like geometry consists of a 750 nm diameter pillar with a 600 nm etch depth. The initial nanojet forms below the zero plane. An approximate diffraction-based interpretation was used in defining the geometry.

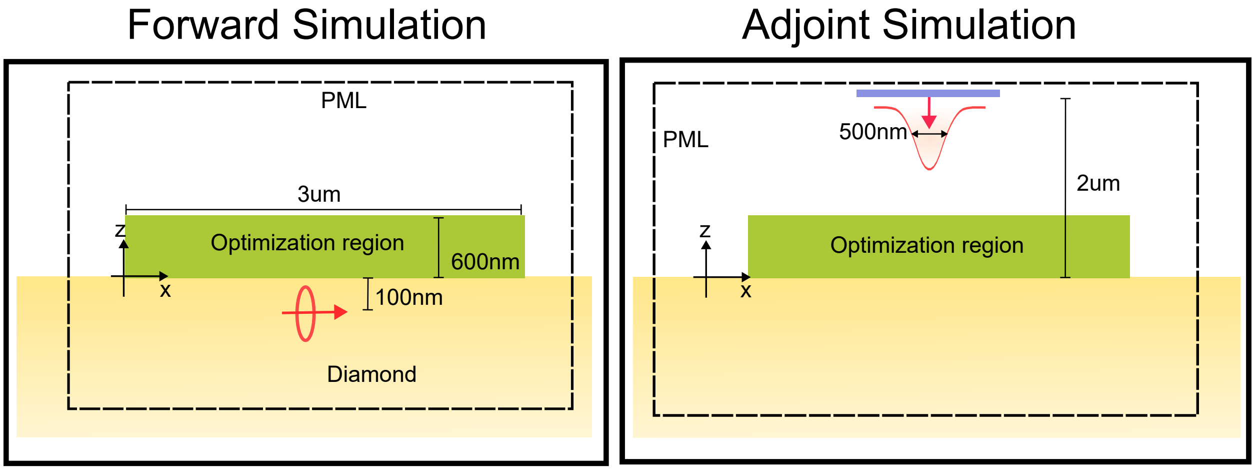

Figure S2: Schematics of the optimization routine. The routine evolves an index profile situated above an NV emitter. In the forward simulation, the light source is modeled as a dipole located at the NV position, approximately 100nm beneath the structure. In the adjoint simulations, the sources are x-polarized Gaussian beams with diffraction-limited spot size, injected from free space toward the structure.

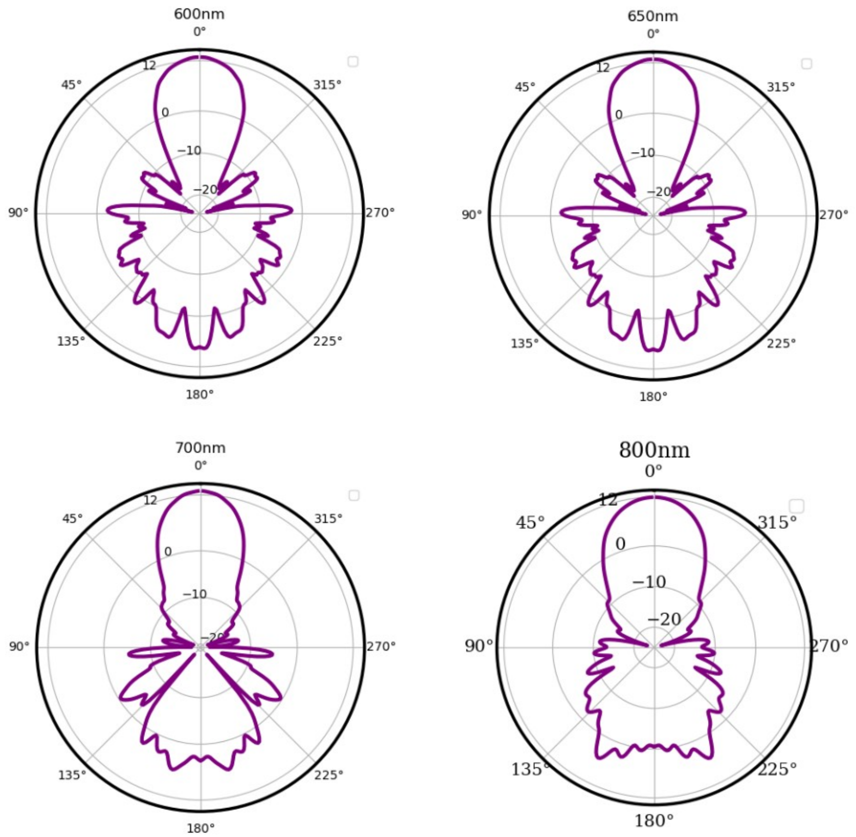

Figure S3: Broadband photon out-coupling from the inverse-designed structure. The radiation pattern of a single NV center within the photonic nanojet mode is presented for three wavelengths across the NV fluorescence emission band in diamond, maintaining high directivity in the upward direction. The directivity values are presented in dB scale.

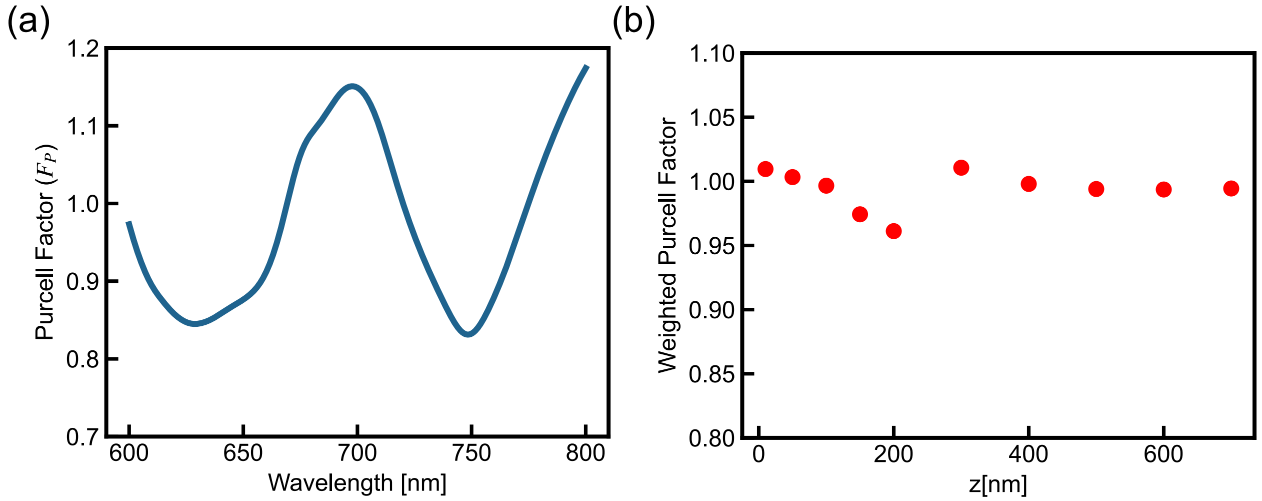

Figure S4: **Simulated Purcell Factor**. (a) Simulated Purcell factor for an x-polarized dipole positioned 100nm below the bare diamond surface. (b) Spectrally weighted Purcell factor as a function of the dipole position within the mode area of the photonic nanojet.

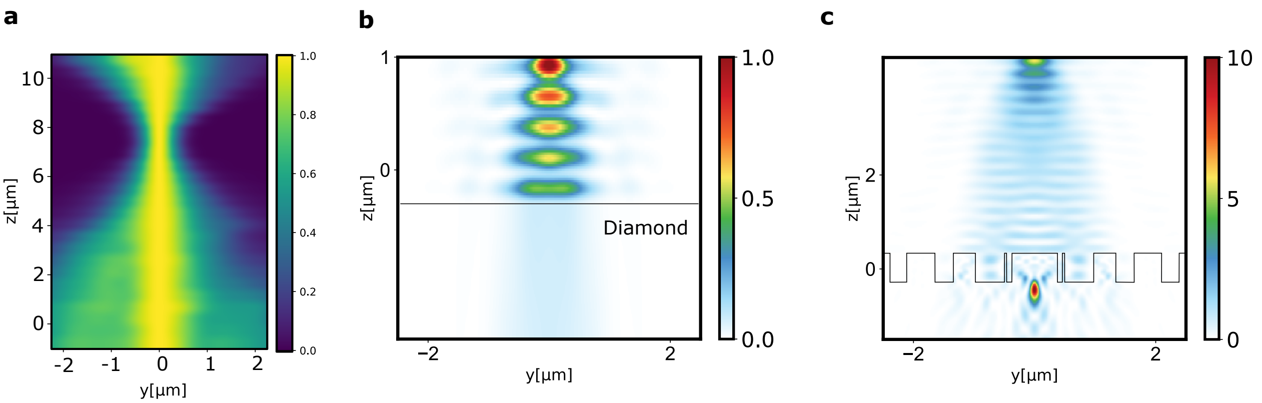

Figure S5: **Excitation simulation based on the measured illumination field profile.** (a) Measured illumination beam profile. (b) Simulated excitation field profile for illumination of bare diamond. The beam waist is positioned close to the surface so that saturation occurs within a reasonable excitation-power range. (c) Simulated excitation field profile using the experimentally measured illumination beam as the input.

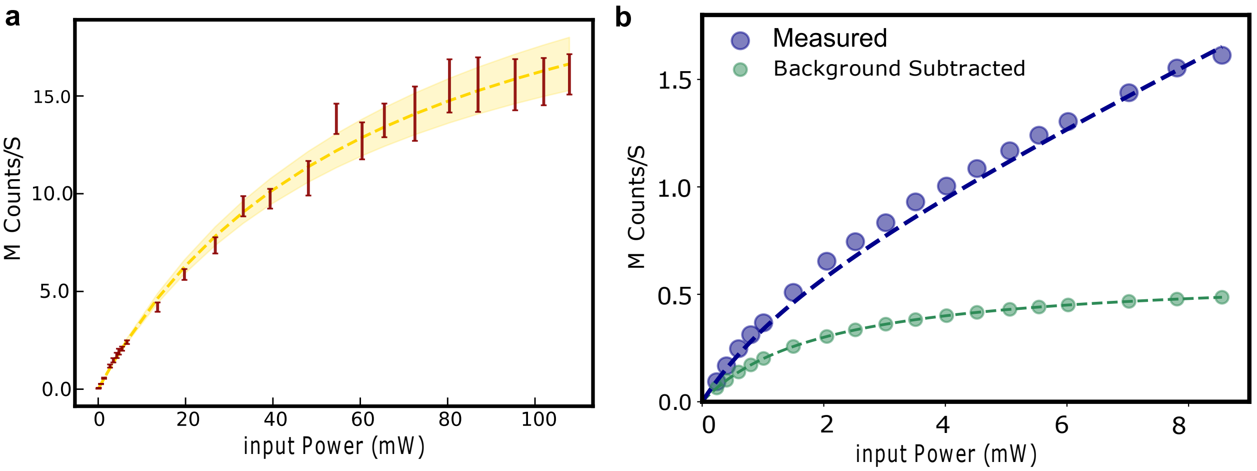

Figure S6: **Saturation curve of background vs. nanojet bright spot.** (a) Measured saturation curve for bare diamond exposed to a focused 532 nm laser. The saturation fit, shown by the yellow region, yields a saturation power of $P_{\mathrm{sat}} = 63.0\,\mathrm{mW} \pm 4\,\mathrm{mW}$. The red error bars indicate variations due to non-uniformity of the counts around the targeted spot. (b) Saturation curve of the bright spot associated with the nanojet device at a height offset of $3\,\mu$m. After background subtraction, the extracted saturation power is $P_{\mathrm{sat}} \approx 0.9\,\mathrm{mW}$.

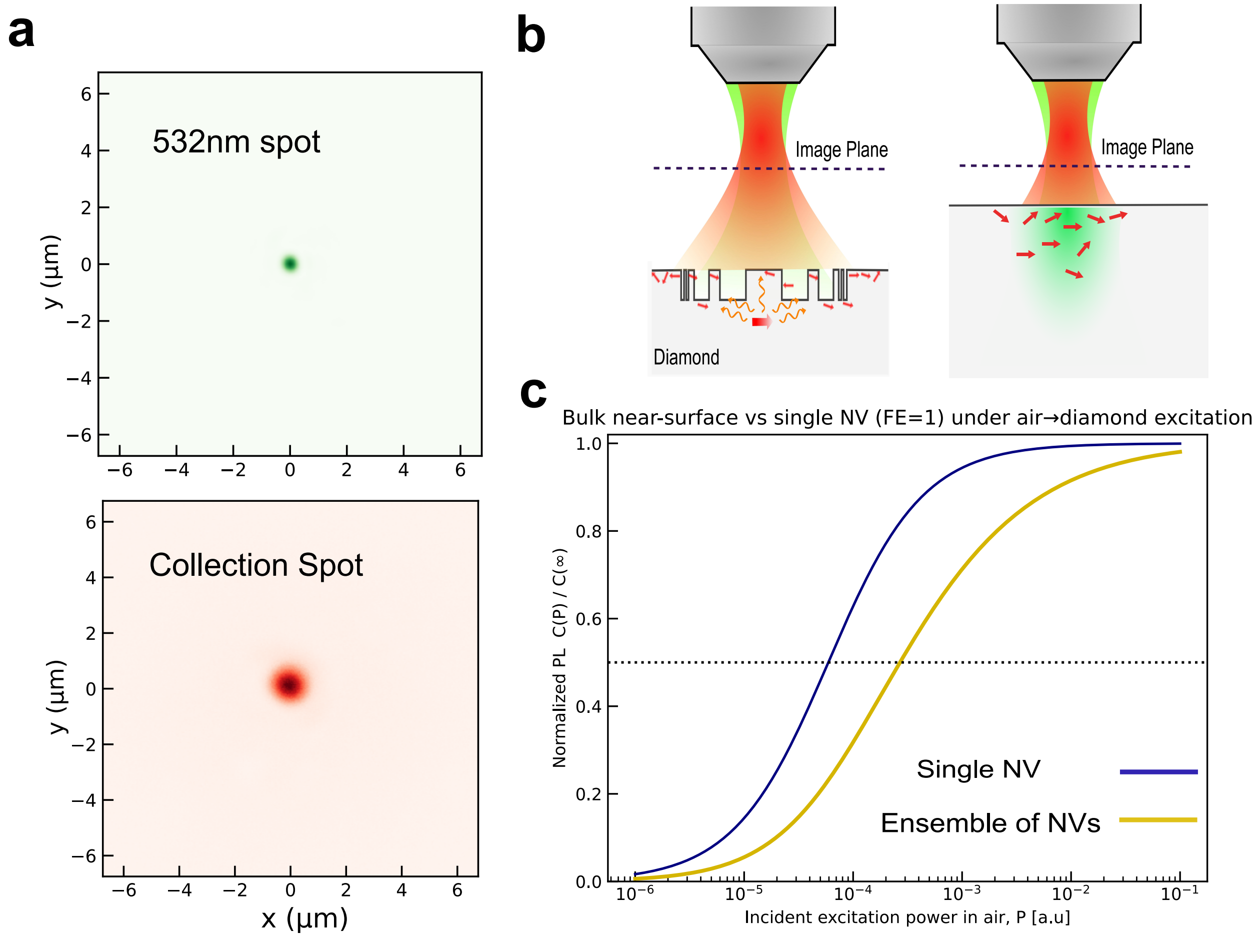

Figure S7: **Saturation behaviour of an ensemble of NV centres in bulk diamond compared to a single NV.** (a) Measured focal spots for the excitation beam at 532 nm, together with the effective detection pinhole size obtained by injecting a 637 nm laser into the collection path. (b) Schematics of the illumination geometry for a nanojet device (left) and bare diamond (right). For the bare diamond case, the Gaussian excitation beam illuminates an ensemble of emitters within its mode profile, with the detected signal weighted by the confocal collection path. (c) Simulated saturation curves for an ensemble of NV centres inside bulk diamond compared to that of a single NV centre excited by a mode with field enhancement of unity.

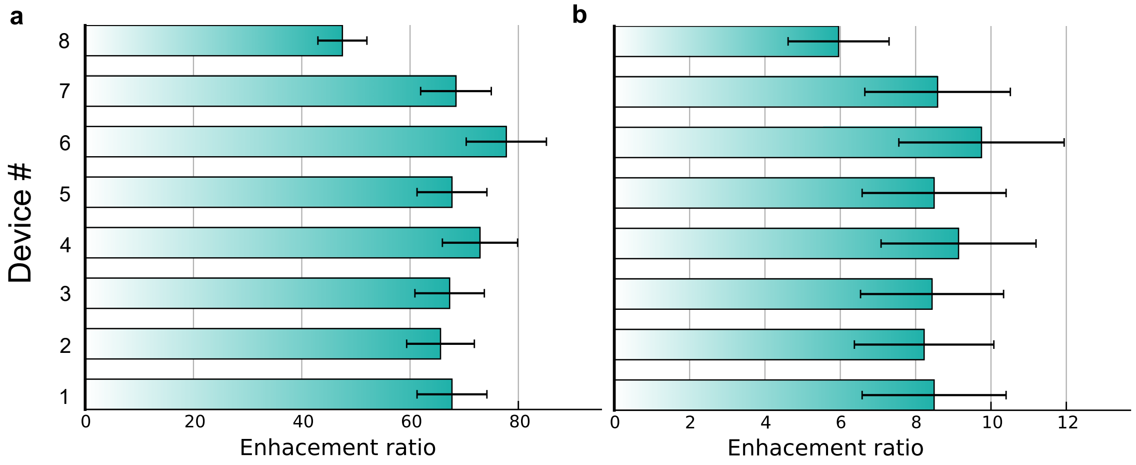

Figure S8: **Excitation enhancement for eight representative devices.** (a) $\mathcal{E}_{\text{raw}}$, obtained by directly comparing the saturation power of bare diamond with that of the nanojet device. (b) $\mathcal{E}_{\text{eff}}$, which relates the field intensity at the nanojet mode to the Gaussian excitation field within its Rayleigh range in air.

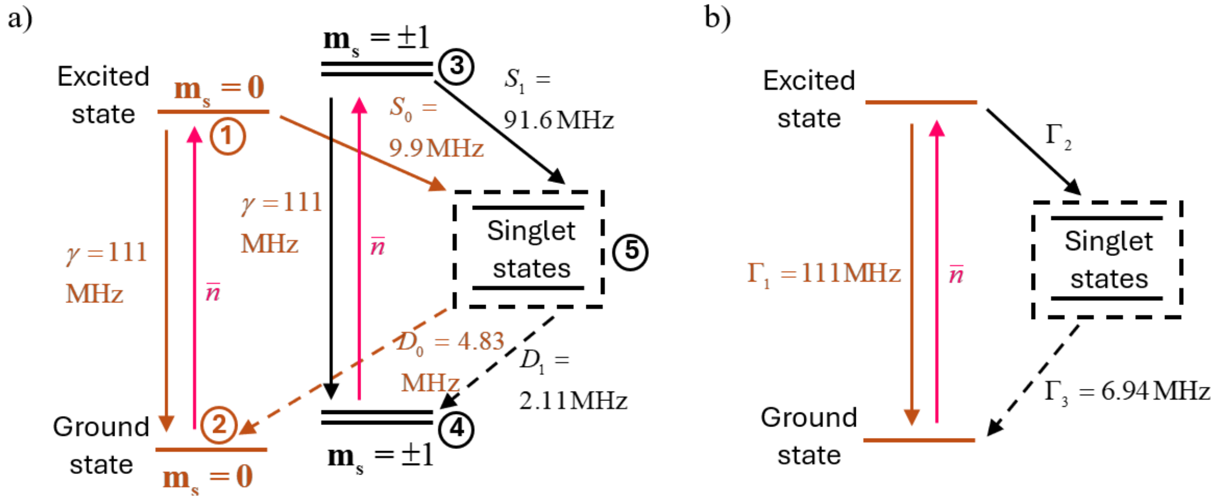

Figure S9: a) Energy level structure of negative NV center at room temperature, b) equivalent 3-level model at steady-state

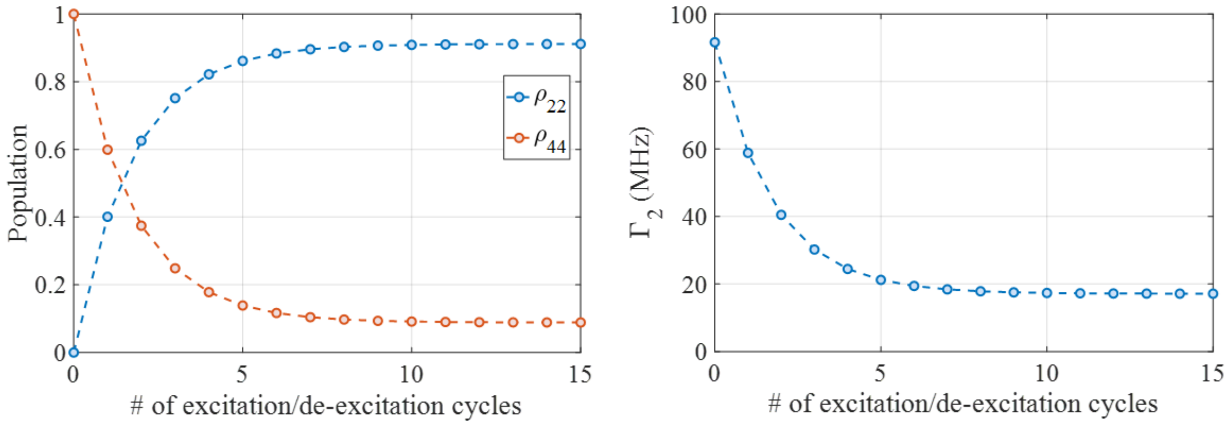

Figure S10: a) Population of different ground states, and b) effective non-radiative decay rate, after multiple excitation and de-excitation cycles of the NV center

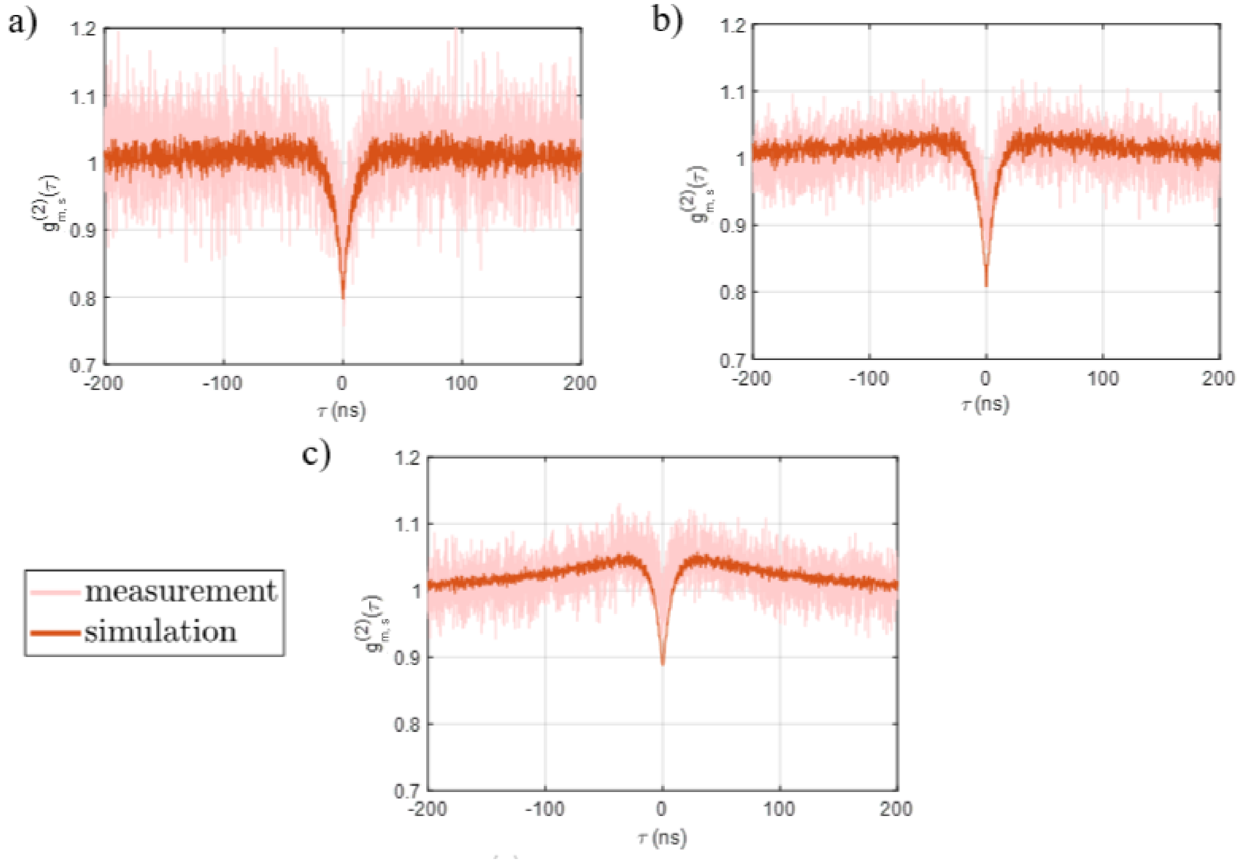

Figure S11: Measured ($g_m^{(2)}(\tau)$) and simulated ($g_s^{(2)}(\tau)$) second-order correlation functions for a) $P = 0.3\,\text{mW}$, b) $P = 0.6\,\text{mW}$, and c) $P = 1.8\,\text{mW}$
